# The Dual-Pseudotyped Lentiviral Vector with VSV-G and Sendai Virus HN Enhances Infection Efficiency through the Synergistic Effect of the Envelope Proteins

**DOI:** 10.3390/v16060827

**Published:** 2024-05-23

**Authors:** Bat-Erdene Jargalsaikhan, Masanaga Muto, Youngeun Been, Shoma Matsumoto, Eiichi Okamura, Tadanobu Takahashi, Yutaka Narimichi, Yuuki Kurebayashi, Hideyuki Takeuchi, Takashi Shinohara, Ryo Yamamoto, Masatsugu Ema

**Affiliations:** 1 Department of Stem Cells and Human Disease Models, Research Center for Animal Life Science, Shiga University of Medical Science, Seta, Tsukinowa-cho, Otsu 520-2192, Japan; baagii@belle.shiga-med.ac.jp (B.-E.J.); smatsu@belle.shiga-med.ac.jp (S.M.); eiokamu@belle.shiga-med.ac.jp (E.O.); 2 Graduate School of Biostudies, Kyoto University, Yoshida-Konoe-cho, Sakyo-ku, Kyoto 606-8501, Japan; been.youngeun.34x@st.kyoto-u.ac.jp; 3 Department of Biochemistry, School of Pharmaceutical Sciences, University of Shizuoka, 52-1 Yada, Suruga-ku, Shizuoka 422-8526, Japan; takahasi@u-shizuoka-ken.ac.jp (T.T.); m19163@u-shizuoka-ken.ac.jp (Y.N.); kurebayashi@u-shizuoka-ken.ac.jp (Y.K.); htakeuchi@u-shizuoka-ken.ac.jp (H.T.); 4 Department of Molecular Genetics, Graduate School of Medicine, Kyoto University, Yoshida-Konoe-cho, Sakyo-ku, Kyoto 606-8501, Japan; tshinoha@virus.kyoto-u.ac.jp; 5 Institute for the Advanced Study of Human Biology (ASHBi), Kyoto University, Yoshida-Konoe-cho, Sakyo-ku, Kyoto 606-8501, Japan; yamamoto.ryo.2c@kyoto-u.ac.jp

**Keywords:** lentiviral vector, gene therapy, Sendai virus, hemagglutinin-neuraminidase, sialic acid, hematopoietic stem cells

## Abstract

A gene delivery system utilizing lentiviral vectors (LVs) requires high transduction efficiency for successful application in human gene therapy. Pseudotyping allows viral tropism to be expanded, widening the usage of LVs. While vesicular stomatitis virus G (VSV-G) single-pseudotyped LVs are commonly used, dual-pseudotyping is less frequently employed because of its increased complexity. In this study, we examined the potential of phenotypically mixed heterologous dual-pseudotyped LVs with VSV-G and Sendai virus hemagglutinin-neuraminidase (SeV-HN) glycoproteins, termed V/HN-LV. Our findings demonstrated the significantly improved transduction efficiency of V/HN-LV in various cell lines of mice, cynomolgus monkeys, and humans compared with LV pseudotyped with VSV-G alone. Notably, V/HN-LV showed higher transduction efficiency in human cells, including hematopoietic stem cells. The efficient incorporation of wild-type SeV-HN into V/HN-LV depended on VSV-G. SeV-HN removed sialic acid from VSV-G, and the desialylation of VSV-G increased V/HN-LV infectivity. Furthermore, V/HN-LV acquired the ability to recognize sialic acid, particularly N-acetylneuraminic acid on the host cell, enhancing LV infectivity. Overall, VSV-G and SeV-HN synergistically improve LV transduction efficiency and broaden its tropism, indicating their potential use in gene delivery.

## 1. Introduction

Gene delivery introduces transgenes through viral or non-viral methods enabling host cells to express proteins or nucleic acids. Recombinant viral vectors have been used in clinics for over 50 years [1,2,3,4], and in gene therapy clinical trials for over 25 years, and comprise integrative and non-integrative vectors, such as lentiviral vectors (LVs), adeno-associated virus vectors, and adenovirus vectors [5,6,7,8,9]. LVs are effective in ex vivo gene therapy for hereditary and acquired diseases because of their long-lasting transgene expression, large transgene packaging capacity, and transduction ability into both dividing and non-dividing cells, and have recently gained regulatory approval [8,10,11,12]. However, the native surface protein of LVs is substituted by another, more suitable, viral envelope protein, using a routine technique known as pseudotyping [13]. 

The LV transduction efficiency relies on the initial interaction between the viral envelope protein and the target receptor on the cell membrane [8]. Thus, pseudotyping can change viral tropism. Vesicular stomatitis virus glycoprotein G (VSV-G) pseudotyping is commonly employed and provides broad tropism enabling LVs to transduce various cell types [13,14,15,16]. Nevertheless, the expression levels of low-density lipoprotein receptor (LDL-R), the primary target for VSV-G [17,18], particularly in resting hematopoietic stem cells (HSCs), T cells, and B cells, is relatively low, which is not ideal for ex vivo LV gene therapy [19,20,21]. Moreover, heterologous (derived from a different virus) viral envelope proteins that are truncated or have engineered cytoplasmic tails have been developed for the effective incorporation of the envelope proteins in lentiviral particle assembly to cope with the challenges of efficient gene transfer into various cell types [19,22,23,24,25,26,27,28,29]. The heterologous envelope proteins that have been applied include BaEV of baboon endogenous retrovirus, RD114 of feline endogenous retrovirus, COCV of cocal vesiculovirus, hemagglutinin and fusion protein (H/F) of measles virus, and fusion and hemagglutinin-neuraminidase protein (F/HN) of Sendai virus [19,22,23,24,25,26,27,28]. 

Phenotypically mixed pseudotyping is a natural occurrence during double viral infection that involves incorporating different viral envelope glycoproteins into single viral particles [30,31,32]. Despite not being as widely used as single-pseudotyped LVs, dual-pseudotyped LVs have certain advantages in specialized applications. Examples of phenotypically unmixed heterologous dual-pseudotyped LVs are the modified F/HN pseudotyped LVs (F/HN-LV) of Sendai virus [28] and the modified H/F pseudotyped LVs (H/F-LV) of measles virus [33]. The modified F/HN-LVs have a high affinity for α2,3 sialylated N-acetyllactosamine (LacNAc), making them ideal candidates for introducing transgenes into airway cells [34,35,36], and the modified H/F-LVs are effective in HSCs, T cells, and B cells [19,37]. Despite the fact that the wild-type F/HN envelope proteins of the Sendai virus could not be incorporated into LVs [28], successful transduction has also been reported in mouse germline stem cells using VSV-G and the Sendai virus wild-type F protein (SeV-F) phenotypically mixed heterologous dual-pseudotyped LVs [38]. The characteristics of VSV-G and wild-type SeV-HN dual-pseudotyped LVs have not been elucidated to date.

LVs continue to play a crucial role in gene delivery as ex vivo gene therapy technologies advance [39]. However, using dual-pseudotyped LVs may pose challenges such as compatibility of the envelope proteins, characterization, and optimization. To address this, we explored using a combination of VSV-G and wild-type HN from Sendai virus to create dual-tropic competence in the LVs. We also attempted to characterize the resulting LVs. Our results showed that the transduction efficiency of V/HN-LV was significantly increased in all of the cell lines tested and the primary cultured cells, especially human cells. We found that VSV-G positively affects the incorporation of wild-type SeV-HN into the V/HN-LV particles, whereas SeV-HN cleaves sialic acid from VSV-G. The desialylated VSV-G increases the infectivity of LVs compared with normal VSV-G. Since SeV-HN recognizes sialylated receptors, we performed V/HN-LV infection on sialic acid-modified cells and revealed that N-acetylneuraminic acid (Neu5Ac) on the host cell membrane is vital to support viral entry. These synergistic actions led to increased infectivity. Furthermore, the SeV-HN envelope protein was optimized for human codons, facilitating the production of the dual-pseudotyped LVs. In summary, the significantly enhanced transduction efficiency of phenotypically mixed heterologous dual-pseudotyped V/HN-LV is due to the functionally broad dual-tropic capability, which widens its potential application for gene delivery.

## 2. Materials and Methods

### 2.1. Cell Culture

HEK293FT, human primary dermal fibroblasts, Caco-2, HeLa, Hep3B, cynomolgus monkey primary dermal fibroblasts, COS-7, and NIH3T3 cells were cultured at 37 °C in a 5% CO_2_ humidified atmosphere in high-glucose Dulbecco’s modified Eagle’s medium (DMEM, 11960-044, Gibco, Grand Island, NY, USA) supplemented with 10% fetal bovine serum (FBS, S1810-500, BioWest, Nuaillé, France), 6 mM L-glutamine (25030-081, Gibco), 1 mM sodium pyruvate (11360-070, Gibco), and 1 × non-essential amino acids (NEAA, 11140-050, Gibco). 

Human trophoblast stem cells (TSCs) were cultured at 37 °C in a 5% CO_2_ humidified atmosphere in DMEM/F-12 GlutaMAX (10565-018, Gibco) supplemented with 0.1 mM 2-mercaptoethanol (M3148, Sigma, Burlington, MA, USA), 0.2% FBS (172012, Sigma), 0.3% bovine serum albumin (BSA, A3311, Sigma), 1% ITS-X supplement (094-06761, Wako, Osaka, Japan), 1.5 µg/mL L-ascorbic acid (013-12061, Wako), 50 ng/mL epidermal growth factor (EGF, 053-07871, Wako), 0.5% penicillin-streptomycin (15140-122, Gibco), 2 μM CHIR99021 (034-23103, Wako), 0.5 μM A83-01 (035-24113, Wako), 1 μM SB431542 (031-24291, Wako), 0.8 mM VPA (227-01071, Wako), and 5 μM Y27632 (331752-47-7, Wako) in a 5 µg/mL collagen IV (354233, Corning Inc, Corning, NY, USA)-coated dish [40].

Mouse embryonic stem cells (mESCs) were maintained at 37 °C in a 5% CO_2_ humidified atmosphere in high-glucose DMEM (08488-55, Nacalai, Kyoto, Japan) supplemented with 20% knockout serum replacement (KSR, 10828-028, Gibco), 1 mM sodium pyruvate (11360-070, Gibco), NEAA (11140-050, Gibco), 0.1 mM 2-mercaptoethanol (198-15781, Wako), penicillin-streptomycin (15140-122, Gibco), mouse leukemia inhibitory factor (LIF, in-house), 1 µM PD0325901 (162-25291, Wako), and 3 μM CHIR99021 (034-23103, Wako) on irradiated mouse embryonic fibroblast (MEF) feeder cells. The culture medium was changed every day. Then, the mESCs were passaged in 0.1% gelatin-coated plates for feeder-free culture expansion [41]. 

Human naïve ESCs were maintained at 37 °C in a 5% O_2_ and 5% CO_2_ humidified atmosphere in a PXGL medium on irradiated mouse MEF feeder cells. ROCK inhibitor Y-27632 (331752-47-7, Wako) was added only during seeding. The culture medium was refreshed every day. The PXGL medium was composed of Ndiff227 (Y40002, Takara Bio, Kusatsu, Japan) medium supplemented with 1 μM PD0325901 (162-25291, Wako), 2 μM XAV939 (575545, Sigma), 2 μM Gö6983 (S2911, Selleck, Houston, TX, USA), and 10 ng/mL human LIF (593906, BioLegend, CA, USA). For feeder-free culture expansion, the human naïve ESCs were seeded in a PXGL medium with Geltrex (10 µg/cm^2^ surface area, A14133-02, Gibco) in a culture plate [42].

The frozen stock of human cord blood samples was thawed and cultured with 1% PSG, 0.01% Solplus, 1% ITS-X, 5 µM of 740Y-P, 0.1 µM of butyzamide, and 1 ng/mL of hFLT3L for four days before LV transduction.

### 2.2. Plasmids

Transfer plasmid CS-CA-NLS-GFP expressing NLS-GFP protein originating from CS-CA-GFP plasmid (RDB05964), packaging plasmids pCAG-HIVgp (RDB04394) encoding HIV1 gag/pol protein and pRSV-Rev expressing HIV1 Rev protein, envelope plasmid pCMV-VSV-G (RDB04392) carrying the gene for vesicular stomatitis virus G glycoprotein (VSV-G), and VSV-G and Rev co-expressing plasmid pCMV-VSV-G-RSV-Rev (RDB04393) were obtained from the RIKEN Institute, Japan. The F and HN proteins were encoded by the wild-type strain of Sendai virus Z, generating plasmids pCAG-F and pCAG-HN, respectively. The Kozak sequence was added in front of HN and human codon-optimized HN, generating plasmids pCAG-kHN and pCAG-khcHN, respectively. The CMAH gene fragment from the green monkey (*Chlorocebus sabaeus*) was subcloned into pPB-CAG-MCS-IRES-Puro plasmid. Plasmid pCAG-hyPBase encoding the hyperactive piggyBac transposase was a gift from the Sanger Institute, United Kingdom.

### 2.3. Lentivirus Production

The HEK293FT producer cells were seeded and allowed to reach approximately 80% confluence, typically 1.5 × 10^6^ cells per 10 cm plate, 2 days before the time of transfection (2.5 × 10^6^ cells per 15 cm plate 3 days before). On the day of transfection, the medium in each 10 cm dish was replaced with 15 mL of fresh medium (45 mL for 15 cm plate). Then, 4–5 h after medium replacement, CS-CA-NLS-GFP, pCAG-HIVgp, pRSV-Rev, pCMV-VSV-G, pCMV-VSV-G-RSV-Rev, pCAG-F, pCAG-HN, pCAG-kHN, pCAG-khcHN, and pCAGGS empty vectors were transfected into the HEK293FT cells using the 25 kDa linear polyethylenimine (25 kDa L-PEI, 23966, Polysciences, Warrington, PA, USA) method at a 5:1 ratio of PEI:DNA. A certain amount of plasmid was mixed in Hanks’ balanced salt solution (HBSS, 09735-75, Nacalai), and PEI was added (Tables S1–S5). The DNA/PEI complex was immediately vortexed for 15 sec and incubated at room temperature for 10 min. The PEI/DNA complex was gently pipetted, added dropwise onto the medium, and mixed by gentle swirling. Then, the medium of the transfected cells was changed after an 18 h incubation. After 52–56 h, the medium was harvested from the transfected cells, then passed through a 0.45-µm Millex-HV PVDF filter (SLHVR33S, Merck Millipore, Burlington, MA, USA), aliquoted, and stored at −150 °C. 

Concentrated LV particles were obtained by ultracentrifugation of the filtered viral medium. Briefly, 8 mL of the medium was carefully added to a 30 mL polypropylene tube (358126, Beckman Coulter, Brea, CA, USA) containing 10 mL of 20% sucrose solution. Then, the samples were placed into a SW 32 Ti rotor and centrifuged at 50,000× *g* for 2 h at 4 °C using an Optima L-90K ultracentrifuge (Beckman Coulter). After the removal of the supernatant, HBSS was added to resuspend the LV pellet. The suspension of concentrated LV particles was mixed by gently pipetting, aliquoted, and stored at −150 °C. The concentration of HIV1 p24 levels was measured by an enzyme-linked immunosorbent assay (ELISA) using a Lenti-X p24 Rapid Titer Kit (632200, Takara Bio) according to the manufacturer’s instruction. The number of LV particles, estimated as 1 ng p24, is equivalent to 1.25 × 10^7^ LV particles.

### 2.4. LV Transduction of HEK293FT Cells and the Biological Titer

To determine the biological titer, HEK293FT cells were trypsinized and resuspended in culture medium, and 2.5 × 10^5^ cells were mixed with LV supernatant in each well of a 24-well plate. After 24 h, the medium of each well was replaced with 0.5 mL of fresh culture medium. Then, 2 days after infection, the cells were washed with PBS and dissociated with TrypLE select (12563-029, Gibco) for 2 min at 37 °C. The trypsinized cells were re-suspended with 2% FBS and filtered through a 35 µM strainer for flow cytometry analysis. The GFP-positive cells were observed using a fluorescence microscope BZ-9000 (Keyence, Osaka, Japan) and were quantified using a FACS Calibur flow cytometer (Becton Dickinson, Franklin Lakes, NJ, USA). Based on the GFP-positive cells between 1.0% and 25.0%, the biological titer of the LV particles was calculated and presented as transduction units per milliliter (TU/mL) using the equation below:

$$TU/mL=\frac{(Initial\;number\;of\;transduced\;cells\times percentage\;of\;{GFP}^{+}\;cells)/100}{Volume\;of\;viral\;medium\;(mL)}$$

### 2.5. LV Transduction of Various Cell Types

The cells were trypsinized and resuspended in their respective culture medium. Then, 2.5–5.0 × 10^4^ cells were mixed with LV particles at an appropriate number of physical particles per cell (PP/cell), and the transduced cells were seeded into a 24-well plate. After 24 h, the medium of each well was replaced with 0.5 mL of fresh culture medium. Finally, 2–4 days after infection, the GFP-positive cells were observed under an inverted fluorescence phase-contrast microscope BZ-9000 and were quantified using a FACS Calibur.

### 2.6. LV Transduction on Desialylated HEK293FT Cells

Endogenous sialyltransferase inhibition assay: HEK293FT cells were treated with 250 µM sialyltransferase inhibitor (STi) (3Fax-Peracetyl Neu5Ac, 566224, Sigma) and maintained for 7 days. For further LV infection experiments, HEK293FT cells (5.0 × 10^4^ cells per/well) were seeded in a 24-well plate and incubated at 37 °C in a 5% CO_2_ humidified atmosphere for 1 day.

Sialidase A treatment assay: HEK293FT cells (5.0 × 10^4^ cells per/well) were seeded in a 24-well plate and incubated at 37 °C in a 5% CO_2_ humidified atmosphere for 1 day. Then, sialidase A (100 mU/mL) (*Arthrobacter ureafaciens*, 10269611001, Roche, Basel, Switzerland) was added to the culture medium and incubated at 37 °C for 2 h. The adherent cells were then carefully washed with basal medium twice.

After the preparation of desialylated cells and non-treated control cells, the culture medium was removed by aspiration, and 0.5 mL of fresh culture medium with V-LV or V/khcHN-LV was added at 800 PP/cell. The medium of each well was replaced with 0.5 mL of fresh culture medium 24 h post-infection. Then, 2 days after infection, GFP-positive cells were observed under an inverted fluorescence phase-contrast microscope BZ-9000 and were quantified using a FACS Calibur.

### 2.7. Desialylation of VSV-G

Desialylation of V-LV: The concentrated LV particles were treated with sialidase A (100 mU/mL) for 1 h at 37 °C, and then LVs were subjected to western blot analysis. 

Functional assay for desialylated V-LV: Unconcentrated V-LV medium was treated with sialidase A (100 mU/mL) for 1 h at 37 °C. Subsequently, 5.0 × 10^4^ HEK293FT cells were transduced with the desialylated V-LV at 800 PP/cell and then seeded into a 24-well plate. After 24 h, the medium in each well was replaced with 0.5 mL of fresh culture medium. Two days after infection, GFP-positive cells were observed under an inverted fluorescence phase-contrast microscope BZ-9000 and were quantified using a FACS Calibur.

### 2.8. Treatment with Neu5Ac and Neu5Gc

HEK293FT cells were maintained in a medium containing Neu5Ac (A1105, TCI Chemicals, Tokyo, Japan) or Neu5Gc (G0336, TCI Chemicals) for 9 days. Then, the Neu5Ac or Neu5Gc-treated HEK293FT cells were washed with PBS, trypsinized, and resuspended in the culture medium. Immediately following this, 5 × 10^4^ of the cells were mixed with LV particles (V-LV or V/HN-LV) at 400 PP/cell and plated in a 24-well plate. The medium of each well was replaced with 0.5 mL of fresh medium at 24 h post-infection. Two days after infection, GFP-positive cells were observed under an inverted fluorescence phase-contrast microscope BZ-9000 and were quantified using a FACS Calibur.

### 2.9. Establishment of CMAH-HEK293FT Cells

HEK293FT cells were seeded and allowed to reach 70% confluence in a 10 cm dish. Four hours before transfection, the medium was replaced with 15.0 mL of fresh medium. Then, PEI-mediated transfection was performed as follows: 16.0 µg of pPB-ITR-cHS4-CAG-CMAH-IP-cSH4-ITR and 0.5 µg of pCAG-hyPBase vector were added to a 1.5 mL Eppendorf tube containing 900 µL of HBSS. Then, 82.5 µg of PEI was added to the DNA complex, and immediately vortexed for 15 sec. The DNA/PEI complex was incubated at room temperature for 10 min. Subsequently, the transfection complex was gently pipetted, added dropwise onto the medium, and mixed by gentle swirling. After 18 h incubation, the medium of the transfected cells was replaced with 10 mL of fresh medium. Two days after transfection, cells were passaged and maintained in a culture medium supplemented with 5 µg/mL of puromycin for 7 days.

### 2.10. Lectin Staining

The cells were fixed with 4% paraformaldehyde (PFA) for 10 min at room temperature and washed three times with PBS. The fixed cells were incubated with 5 µg/mL of biotinylated lectin SNA (B-1305, Vector Laboratories, Burlingame, CA, USA), MALI (B-1315, Vector Laboratories), and MALII (B-1265, Vector Laboratories) in PBS at room temperature for 1 h, followed by three washes with PBS. The cells were then incubated with GFP-labeled secondary antibody (Alexa fluor™ 488 streptavidin conjugated, 1:1000 dilution, S11223, Invitrogen, Carlsbad, CA, USA) in PBS at 4 °C for 1 h, followed by three washes with PBS. Then, cells were incubated with Hoechst (1:1000 dilution, H3570, Invitrogen) in PBS at room temperature for 15 min and washed once with PBS. Immunofluorescence images were recorded using an inverted fluorescence phase-contrast microscope BZ-9000.

### 2.11. Immunostaining

The cells were fixed with 4% PFA for 10 min at room temperature, then washed three times with PBS. The fixed cells were incubated with chicken anti-Neu5Gc antibody (1:500 dilution, 146903, BioLegend) in PBS at 4 °C for 1 h, followed by three washes with PBS. The cells were then incubated with GFP-labeled secondary antibody (Alexa fluor™ Plus 488 Goat anti-chicken, 1:500 dilution, A32931, Invitrogen) in 1 × PBS at 4 °C for 1 h, followed by three washes with PBS. Then, cells were incubated with Hoechst (1:1000 dilution, Invitrogen) in PBS at room temperature for 15 min and washed once with PBS. Immunofluorescence images were recorded using an inverted fluorescence phase-contrast microscope BZ-9000.

### 2.12. Flow Cytometry

The cells were washed with PBS and digested with TrypLE select (12563-029, Gibco) for 2–10 min at 37 °C. Then, the trypsinized cells were re-suspended with 2% FBS and filtered through a 35 µM strainer. The number of GFP-positive cells was determined using FACS Calibur. The main cell population was gated from a Forward Scatter (FSC-H) versus a Side Scatter (SSC-H) plot. Transduced GFP-positive cells were detected just outside of the negative cell population in the FL1-H (530 nm) versus FL2-H (585 nm) plot. The data were analyzed using FlowJo 10.1 software (BD Life Sciences, Ashland, OR, USA).

### 2.13. Western Blot Analysis

Concentrated LV particles were lysed with SDS sample buffer (final concentration: 2% SDS, 10% glycerol, 0.005% bromophenol blue, 62.5 mM Tris, pH 6.8, and 2.5% 2-mercaptoethanol). The lysates were boiled at 95 °C for 5 min, then cooled to room temperature. The lysates, equivalent to 7.5 ng of p24 by ELISA, were subjected to 12.5% SDS-polyacrylamide gel electrophoresis (PAGE) (SuperSep™ Ace, 196-14981, Wako) and transferred to a polyvinylidene difluoride membrane (PVDF, IPVH00010, Merck Millipore). The PVDF membrane was then blocked with 5% skim milk (31149-75, Nacalai) in Tris-buffered saline with 0.1% Tween-20 (TBST) for 1 h at room temperature. The membrane was incubated with primary antibody against VSV-G (P5D4, SAB4200695, Sigma), whole SeV (PD029 MBL Life Science, Tokyo, Japan), or HIV p24 (P131, Ab32352, Abcam, Cambridge, UK) at 4 °C overnight, then horseradish peroxidase (HRP)-conjugated secondary antibody for 1 h. Finally, the membrane was incubated with HRP substrate (SuperSignal™ West Pico PLUS, 34577, Thermo Fisher Scientific) for 2 min, and the signal was detected using the luminescent image analyzer ImageQuant LAS 4000 mini (Fujifilm, Tokyo, Japan). Band intensities were measured using ImageJ 1.54 software (National Institutes of Health, Bethesda MD, USA).

### 2.14. Statistical Analysis

All statistical analyses were performed using Prism 9.5.1 software (GraphPad Software, San Deigo, CA, USA). All data are expressed as the mean ± standard deviation (SD) with n ≥ 3 independent replicates per condition. Statistical significance was determined using a two-tailed *t*-test and ordinary one-way or two-way ANOVA followed by Tukey post-hoc tests, as indicated in the figure legend. *p* values ≤ 0.05 were considered statistically significant and are presented in each figure legend.

## 3. Results

### 3.1. LVs Pseudotyped with VSV-G and Wild-Type SeV Envelope Proteins

**Figure 1:**
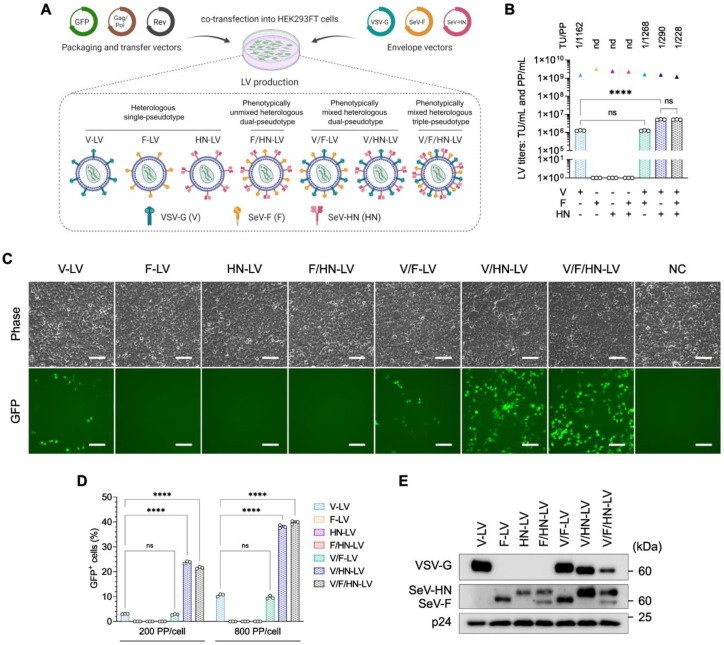
Increased transduction efficiency of LVs pseudotyped with VSV-G and SeV-HN in HEK293FT cells. (**A**) Schematic diagram of LV pseudotyping with the VSV-G and SeV envelope proteins. Seven types of LV particles were produced using transfer, packaging, Rev, and envelope plasmids in a 4:2:1:1 ratio, as detailed in Table S1. (**B**) The biological and physical titers of LVs. The bar graphs represent TU/mL, and the triangles represent PP/mL. The ratio of TU to PP is shown above each set of LV titers. (**C**) The expression level of GFP was observed by fluorescence microscopy 48 h after LV transduction at 200 PP/cell in HEK293FT cells. Scale bar, 100 µm. (**D**) HEK293FT cells were infected with LV particles at the indicated PP/cell and the percentage of GFP-positive cells was determined at 2 days post-infection by flow cytometry. (**E**) Ultracentrifuged LV particles were subjected to western blotting using VSV-G, whole SeV, and HIV1 p24 antibodies. Data are expressed as the mean ± SD with technical replicates (n = 3). Ordinary one-way ANOVA with Tukey post-hoc test was conducted for (**B**) and ordinary two-way ANOVA with Tukey post-hoc test was conducted for (**D**). **** *p* < 0.0001; ns, not significant; nd, not detected.

The phenotypically mixed pseudotyping of vesicular stomatitis virus (VSV) naturally appears when the host cells are co-infected with VSV and viruses from the Paramyxoviridae family, such as Sendai virus [30,43]. Even though truncated SeV-F and engineered SeV-HN were designed for LVs [28], the natural phenomenon suggests the possibility of dual-pseudotyping of the LVs with the VSV-G and wild-type SeV envelope proteins. Shinohara et al. (2020) demonstrated the dual-pseudotyping of LVs using VSV-G and wild-type SeV-F glycoproteins [38]. The difference between these envelope proteins is illustrated in the Supplementary Figure (Figure S1A). We employed a human immunodeficiency virus 1 (HIV1)-based third-generational self-inactivating LV system and produced seven types of LV particles, which encoded enhanced green fluorescent protein (GFP), using a combination of VSV-G and wild-type SeV envelope proteins (Figure 1A, Table S1).

The biological titer [transduction units (TU)/mL] and physical titer [physical particles (PP)/mL] were determined and compared (Figure 1B). The production of V-LV and V/F-LV created average biological titers of 1.26 × 10^6^ TU/mL and 1.25 × 10^6^ TU/mL, respectively. Although the physical titers of the LV particles were comparable, ranging between 1.17 × 10^9^ PP/mL and 1.59 × 10^9^ PP/mL, the LVs pseudotyped with F and/or HN without VSV-G (F-LV, HN-LV, and F/HN-LV) were not infectious in HEK293FT cells, and neither the biological titer nor the TU/PP ratio were able to be calculated. In addition, the LVs that were pseudotyped with SeV-HN along with VSV-G showed a significant 4-fold increase compared with the other pseudotyped LVs, with titers of 5.19 × 10^6^ TU/mL for V/HN-LV and 5.15 × 10^6^ TU/mL for V/F/HN-LV. When the pseudotyping of VSV-G and SeV-HN was combined, the TU/PP ratio was increased 4-fold compared with the V and V/F pseudotyped LVs, as shown in Figure 1B.

To evaluate the infectivity of these LV particles, HEK293FT cells were infected with an equal number of PPs per cell. The effect of V/F-LV was comparable to that of V-LV. Remarkably, the transduction efficiency of LVs pseudotyped with VSV-G and SeV-HN (V/HN-LV and V/F/HN-LV) was dramatically increased compared with other pseudotyped LVs (Figure 1C,D and Figure S1B). Thus, we conducted a western blot analysis to determine whether the envelope proteins were incorporated into LVs. We did observe a difference in the intensity of SeV-F and SeV-HN proteins between the LV particles enveloped with VSV-G (V/F-LV and V/HN-LV) and without VSV-G (F-LV, HN-LV, and F/HN-LV). By contrast, when all of the envelope proteins were combined, the incorporation efficiency was decreased (Figure 1E). These findings suggested the positive effects of VSV-G on the incorporation of wild-type SeV-F or SeV-HN in the LV particles. In summary, the wild-type SeV envelope proteins F or HN had been correctly incorporated within the LV particles along with VSV-G, and V/HN-LV drastically improved the transduction efficiency of HEK293FT cells.

### 3.2. Transduction Efficiency of LVs Pseudotyped with VSV-G and SeV Envelope Proteins in Various Cell Types

**Figure 2:**
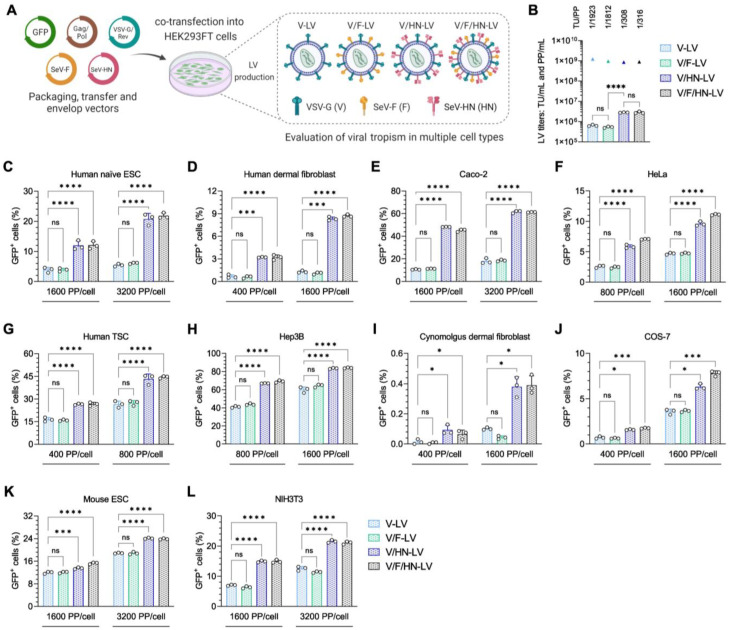
Viral tropism of LVs with VSV-G and SeV envelope proteins. (**A**) LV particles used in the tropism assay were produced using transfer, packaging, VSV-G/Rev, SeV-F, and/or SeV-HN envelope plasmids in a 2:1:1:1 ratio, as detailed in Table S2. (**B**) The biological and physical titers of LVs. The bar graphs represent TU/mL, and the triangles represent PP/mL. The ratio of TU to PP is shown above each set of LV titers. (**C**–**H**) Human cells: (**C**) human naïve embryonic stem cells (ESCs), (**D**) human primary dermal fibroblast cells, (**E**) colorectal adenocarcinoma cell line Caco-2, (**F**) human cervical carcinoma derived cell line HeLa, (**G**) human trophoblast stem cells (TSCs), and (**H**) human hepatocellular carcinoma cell line Hep3B; (**I**,**J**) monkey cells: (**I**) cynomolgus monkey primary dermal fibroblast cells and (**J**) African green monkey kidney fibroblast-like cell line COS-7; and (**K**,**L**) mouse cells: (**K**) mouse embryonic stem cells (ESCs) and (**L**) mouse embryonic fibroblast cell line NIH3T3, were transduced with four types of LV particles at the indicated PP/cell. The infection efficiency was measured 2–4 days post-infection by counting GFP-positive cells via flow cytometry. Data are expressed as the mean ± SD with technical replicates (n = 3). Ordinary one-way ANOVA with Tukey post-hoc test was conducted for (**B**) and ordinary two-way ANOVA with Tukey post-hoc test was conducted for (**C**–**L**). **** *p* < 0.0001, *** *p* < 0.001, and * *p* < 0.05; ns, not significant.

Since VSV-G and wild-type SeV-HN dual-pseudotyping increased the transduction efficiency of LVs on HEK293FT cells, we next explored the transduction efficiency of V/HN-LV particles (including V-LV, V/F-LV, V/HN-LV, and V/F/HN-LV) on a wide range of mammalian cell types (Figure 2A, Table S2).

In this experiment, the LV production of V and V/F pseudotypes yielded biological titers of 6.37 × 10^5^ TU/mL and 5.28 × 10^5^ TU/mL, respectively. Moreover, an approximate 4.5-fold improvement in functional titer was observed for V/HN-LV and V/F/HN-LV production, generating 2.80 × 10^6^ TU/mL and 2.83 × 10^6^ TU/mL, respectively. The physical titers of the LV particles were similar. Whereas, the addition of SeV-HN pseudotyping for LVs led to a 6-fold increase in the TU/PP ratio when compared with other pseudotyped LVs (Figure 2B). For the cell tropism assay, 2.5–5.0 × 10^4^ cells were infected with the LV particles. The number of LV particles was calculated as PP/cell and was equal in the LV groups. The human cell lines utilized for LV transduction were human naïve embryonic stem cells (ESCs), primary dermal fibroblast cells, Caco-2 cells, HeLa cells, trophoblast stem cells (TSCs), and Hep3B cells (Figure 2C–H); the cynomolgus monkey cell lines tested were dermal fibroblast cells and COS-7 cells (Figure 2I,J); and the mouse cell lines tested were ES cells and NIH3T3 cells (Figure 2K,L). Quantification of the percentage of GFP-positive cells revealed that the transduction efficiencies of V/HN-LV and V/F/HN-LV were significantly increased in all of the cell lines and primary cultured cells (Figure 2C–L and Figure S2). The results showed that VSV-G and wild-type SeV-HN phenotypically mixed heterologous dual-pseudotyping expands the cell tropism of LVs and is useful for the generation of transgenic cell lines in the field of basic research.

### 3.3. Balance of VSV-G and SeV-HN Protein in the LV Particles Determines the Viral Transduction Efficiency

**Figure 3:**
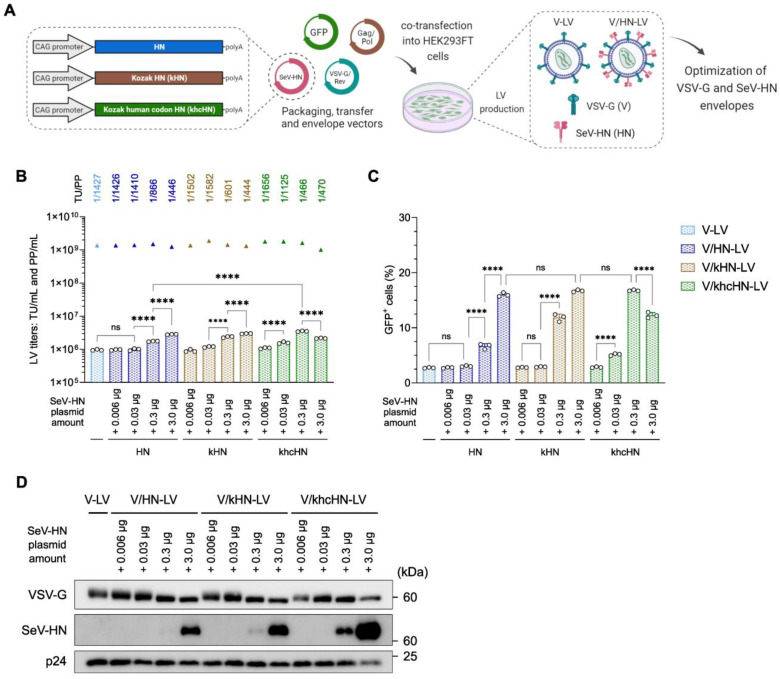
Relationship between the infection efficiency and the amount of SeV-HN protein in the V/HN-LV particles. (**A**) Schematic diagram of the production of LV particles using the modified SeV-HN-expressing plasmids. A 2:1:1 ratio of transfer, packaging, and VSV-G/Rev plasmids, and variable amounts of SeV-HN plasmids (unmodified HN, Kozak HN, or Kozak human codon-optimized HN), were used for the optimization of dual-pseudotyped LV particles. (**B**) The biological and physical titers of LVs. The bar graphs represent TU/mL, and the triangles represent PP/mL. The ratio of TU to PP is shown above each set of LV titers. (**C**) HEK293FT cells were infected with LV particles at 200 PP/cell, and the infection efficiency was determined by flow cytometry 2 days after transduction. (**D**) Ultracentrifuged LV particles were subjected to western blotting using VSV-G, whole SeV, and HIV1 p24 antibodies. Data are expressed as the mean ± SD with technical replicates (n = 3) and ordinary one-way ANOVA with Tukey post-hoc test. **** *p* < 0.0001; ns, not significant.

Optimizing the dual-pseudotyped vector is crucial and requires careful balancing of the envelope proteins in the viral particles. There is also a technical concern that the addition of SeV-HN plasmid DNA increases the total amount of DNA for LV production. Codon optimization of the viral envelope protein significantly reduces plasmid consumption [44]. Therefore, we attempted to minimize the SeV-HN plasmid vector and constructed HN with the Kozak sequence (kHN) and human codon-optimized HN with the Kozak sequence (khcHN) to increase the levels of SeV-HN envelope protein in the LV particles (Figure 3A).

We used different amounts (0.006, 0.03, 0.3, and 3.0 µg) of the three types of plasmids expressing HN for transfection (Table S3). The addition of these HN plasmids did not dramatically affect their physical titers, which ranged between 1.01 × 10^9^ and 1.88 × 10^9^ PP/mL. The production of V-LV, similarly to that described above, generated an average biological titer of approximately 1.0 × 10^6^ TU/mL, which was almost identical to the LV production with the addition of 0.006 µg and 0.03 µg of HN plasmids. Furthermore, a 1.8–3.7-fold significant improvement in biological titer was detected for LV production using 0.3 µg and 3.0 µg of HN plasmids, generating a maximum average titer of approximately 3.49 × 10^6^ TU/mL for the 0.3 µg khcHN plasmid. The TU/PP ratio increased in a dose-dependent manner with additional HN plasmids and reached an increment of approximately 3-fold compared with V-LV (Figure 3B). The viral infection assay was performed as described above. The addition of 0.006 µg and 0.03 µg of kHN and khcHN plasmids did not affect the infection efficiency compared with the unmodified HN plasmid. In the case of 0.3 µg of plasmid, the viral infectivity of the LVs utilizing the kHN plasmid was increased, and human codon optimization with the Kozak sequence showed the most effective transduction, with a rate of 3-fold compared with unmodified HN, even though the same number of LV particles were utilized (Figure 3C and Figure S3A).

**Figure 4:**
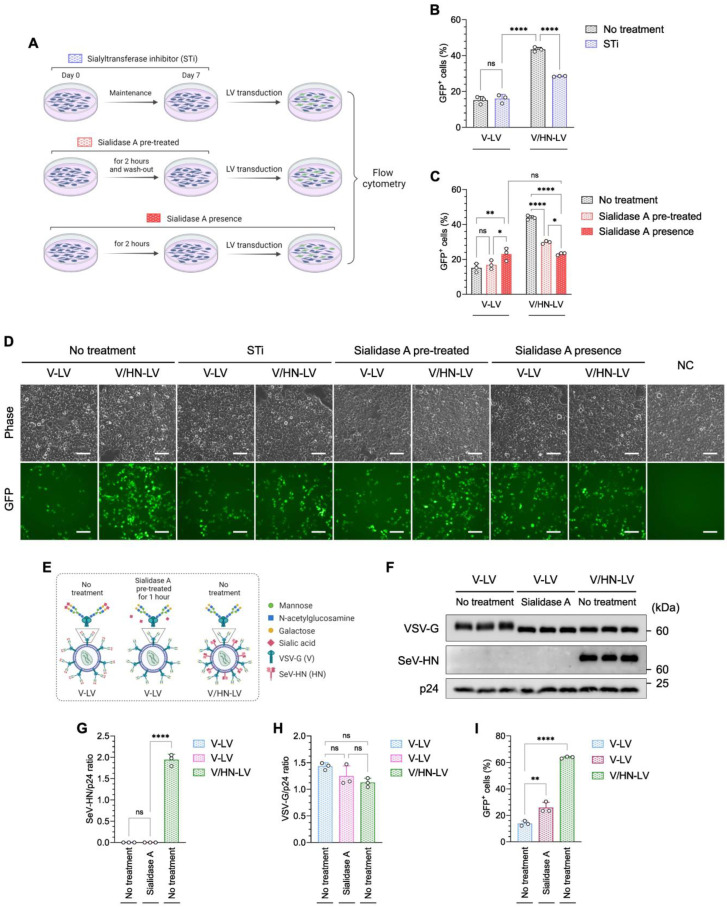
V/HN-LV recognizes the sialylated receptor on the host cell membrane. (**A**) Schematic diagram showing investigation of the importance of sialic acid on the host cell membrane during V/HN-LV infection using (**B**) a sialyltransferase inhibitor (STi) and (**C**) sialidase A treatment. The transduction efficiency of LV particles was determined by counting GFP-positive cells using flow cytometry 48 h after infection at 800 PP/cell. (**D**) Phase contrast and GFP fluorescence images 2 days after infection in the experimental groups. (**E**) Schematic illustration shows the glycan structure of VSV-G in the V-LV and its desialylation by sialidase A. In V/HN-LV, SeV-HN cleaves sialic acid from VSV-G. (**F**) The LV particles obtained by ultracentrifugation of the viral supernatant were treated with sialidase A and subjected to western blotting using VSV-G, whole SeV, and HIV1 p24 antibodies. Untreated V-LV and V/HN-LV were included as negative and positive controls, respectively. (**G**,**H**) Summary graphs of the SeV-HN/p24 and VSV-G/p24 ratios. (**I**) Functional assay of the desialylated V-LV particles at 800 PP/cell. Scale bar, 100 µm. Data are expressed as the mean ± SD with biological replicates (n = 3). Ordinary two-way ANOVA with Tukey post-hoc test was conducted for (**B**,**C**) and ordinary one-way ANOVA with Tukey post-hoc test was conducted for (**G**–**I**). **** *p* < 0.0001, ** *p* < 0.01, and * *p* < 0.05; ns, not significant.

**Figure 5:**
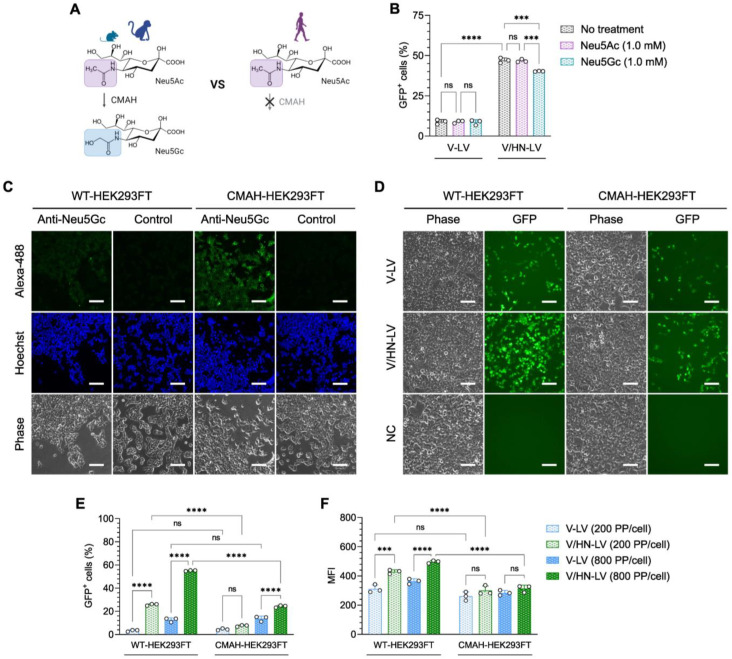
N-glycolylneuraminic acid (Neu5Gc) inhibits V/HN-LV infection. (**A**) The CMAH enzyme converts CMP-Neu5Ac to CMP-Neu5Gc and is a pseudogene in humans and has thereby lost its activity. (**B**) HEK293FT cells were infected with LV particles at 400 PP/cell after feeding with 1.0 mM Neu5Ac or Neu5Gc for 9 days. The GFP-positive cells were evaluated by flow cytometry 2 days after transduction. (**C**) Neu5Gc expression was stained on WT-HEK293FT and CMAH-HEK293FT cells using an anti-Neu5Gc antibody. (**D**,**E**) The infection efficacy of LV particles on WT-HEK293FT and CMAH-HEK293FT cells was evaluated by (**D**) microscopic observation 48 h post-infection at 800 PP/cell and (**E**) flow cytometry analyses 48 h post-infection at the indicated PP/cell. (**F**) Median fluorescence intensity (MFI) was determined by flow cytometry. Scale bar, 100 µm. Data are expressed as the mean ± SD with biological replicates (n = 3) and ordinary two-way ANOVA with Tukey post-hoc test was conducted. **** *p* < 0.0001 and *** *p* < 0.001; ns, not significant.

Interestingly, although western blotting analysis revealed the highest amount of SeV-HN protein in LV particles utilizing 3.0 µg of khcHN plasmids, its infectivity was significantly reduced compared with LVs utilizing 0.3 µg of khcHN plasmids, indicating that a higher level of expression of SeV-HN protein on the viral particle does not increase the infection efficiency (Figure 3C,D). Similar protein expression levels of VSV-G and SeV-HN were shown with 3.0 µg of HN, 3.0 µg of kHN, and 0.3 µg of khcHN, which resulted in a peak level of infectivity (Figure 3D). Based on the optimization, the V/HN-LV particles utilized in the experiments in Figure 4 and Figure 5 were produced with the addition of 0.1 unit of khcHN plasmid, as shown in Table S4. We confirmed that the TU/PP ratio of V/HN-LV was increased approximately 5-fold compared with that of V-LV particles (Figure S3B). Collectively, these results revealed that an appropriate balance of VSV-G and SeV-HN envelope proteins in the viral particles ensures maximal infectivity for the dual-pseudotyped LVs, and the human codon-optimized plasmid vector of SeV-HN envelope protein facilitates its production.

### 3.4. Sialic Acids Are Vital for the Infection Efficiency of V/HN Dual-Pseudotyped LVs

The V/HN-LVs are expected to have dual-tropic competence, because VSV-G and SeV-HN utilize the LDL-R family and sialylated receptors to enter host cells, respectively [18,45]. The envelope protein HN found in viruses belonging to the Paramyxoviridae family has multiple functions: it recognizes sialylated receptors on the cell surface to attach to cells, it promotes fusion activity of the F protein to enter cells, and it removes sialic acid from progeny viruses to prevent self-aggregation [46,47]. We thus hypothesized that SeV-HN in the V/HN-LVs might remove sialic acid from VSV-G in addition to recognizing sialylated receptors on the host. To better characterize the V/HN-LVs, we investigated the implications of terminal sialic acid on host cells and viral particles. We treated HEK293FT cells with a sialyltransferase inhibitor (STi) or sialidase A, which removes terminal sialic acids from the cell surface, then conducted LV transduction (Figure 4A–D and Figure S4A).

Desialylation of the cells was confirmed by lectin staining (Figure S4B,C). The infectivity of V-LV was not altered in STi-treated cells, whereas the GFP-positive rate was significantly decreased when V/HN-LV was added to the cells compared with the no-treatment control (Figure 4B,D). Moreover, the LV infectivity pattern with sialidase A pre-treated cells was similar to that of STi-treated cells (Figure 4C,D), indicating that the SeV-HN in V/HN-LV recognizes sialic acid on the host cells. Interestingly, the infection efficiency was similar between V-LV and V/HN-LV particles in the presence of sialidase A, which was higher than that of V-LV and lower than that of V/HN-LV particles in comparison with the sialidase A pre-treated groups (Figure 4C,D).

Next, we performed a desialylation assay on V-LV particles (Figure 4E–I and Figure S5A). Western blot analysis showed that the band correlating to VSV-G was broader, indicating post-translational modification, and shifted to a lower molecular weight in the V/HN-LV and sialidase A-treated V-LV compared with the no-treatment control. The molecular weights of VSV-G from the V/HN-LV and the sialidase A-treated V-LV particles were comparable (Figure 4F). The amount of VSV-G protein was not affected by sialidase treatment (Figure 4G,H). The infectivity of sialidase A-treated V-LV particles was significantly increased compared with that of the non-treated V-LV particles (Figure 4I and Figure S5B). These results demonstrated that the neuraminidase domain of SeV-HN in V/HN-LV cleaves sialic acid from VSV-G protein on the viral particles, also enhancing infectivity. Taken together, we confirmed that the neuraminidase domain of SeV-HN cleaves the sialic acid of VSV-G protein on the viral particles and the SeV-HN retains the ability to recognize sialic acid, thereby synergistically improving viral infectivity.

### 3.5. V/HN-LV Particles Enter Cells by Utilizing the Neu5Ac Expressed on the Host Cells

SeV-HN glycoprotein generally recognizes Neu5Ac and Neu5Gc on the cell surface, which are common types of sialic acid linked to the subterminal galactose of glycoproteins and glycolipids in mammalian cells [45]. As a result of a dysfunctional cytidine monophospho-N-acetylneuraminic acid hydroxylase (CMAH) gene, which is required for converting CMP-Neu5Ac to CMP-Neu5Gc, human cells cannot synthesize Neu5Gc [48,49] (Figure 5A).

However, Neu5Ac and Neu5Gc enter cellular lysosomes via the endocytic pathway from dietary sources [50]. The lysosomal transporter releases free sialic acids into the cytosol. These are then converted to CMP-Neu5Ac or CMP-Neu5Gc in the nucleus and recycled for sialylation reactions in the Golgi. Finally, sialylated glycoproteins are transported to the cell surface [51,52]. Therefore, to confirm the type of sialic acid utilized by V/HN-LVs to enter cells, we performed an LV infection assay in HEK293FT cells fed sialic acid, Neu5Ac, or Neu5Gc (Figure S6A). We found that the V/HN-LV transduction efficiency was decreased in the Neu5Gc-fed cells (Figure 5B and Figure S6B).

Additionally, we established a HEK293FT cell line that stably overexpressed the monkey CMAH gene (CMAH-HEK293FT), and its function was confirmed in the cells by Neu5Gc staining (Figure 5C). The infection efficiency of V/HN-LV was remarkably decreased in the CMAH-HEK293FT cells compared with parental HEK293FT cells (Figure 5D,E), confirming the LV infectivity data in the Neu5Gc-fed cells. Even for the median fluorescence intensity (MFI), a reduction pattern was detected in CMAH-HEK293FT cells (Figure 5F). There was no difference between the conditions examined for V-LV particles (Figure 5B–F). Ultimately, our findings revealed that the infectivity of V/HN-LV is inhibited by Neu5Gc in human cells, suggesting the preferential utilization of SeV-HN proteins and Neu5Ac for viral entry.

### 3.6. Effectiveness of the V/HN-LV Particles in Human Hematopoietic Stem and Progenitor Cells

**Figure 6:**
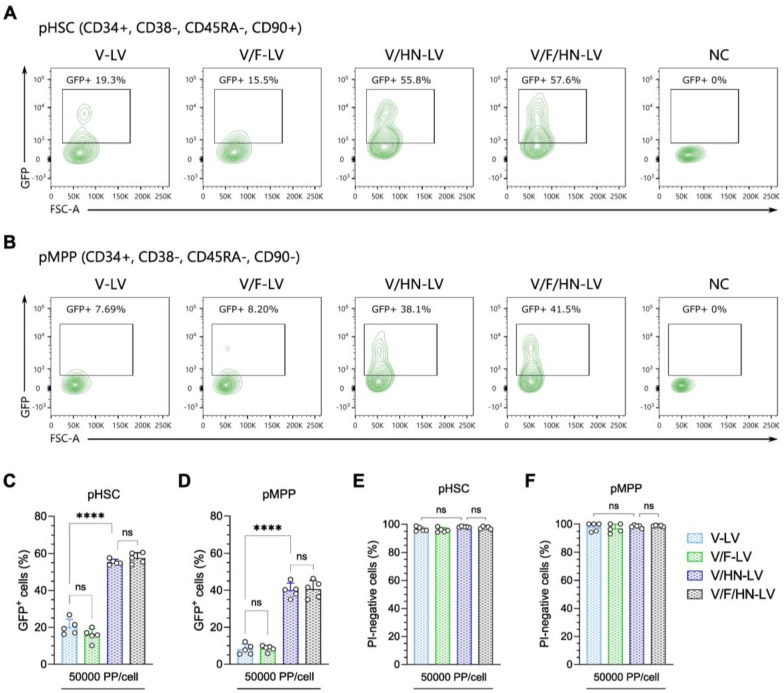
Improvement in the transduction efficiency of VSV-G and SeV-HN pseudotyped LVs in human hematopoietic stem and progenitor cells. (**A**,**B**) Representative flow cytometry plots of the GFP-positive rate in (**A**) with the human phenotypic hematopoietic stem cell (pHSC) population, defined as CD34^+^, CD38−, CD45RA−, and CD90^+^, and (**B**) with the phenotypic multipotent progenitor (pMPP) cell population, defined as CD34^+^, CD38−, CD45RA−, and CD90−. Human cord blood cells were infected with the four types of concentrated LV particles at 50,000 PP/cell, with production as shown in Table S5. The GFP-positive cells (**C**,**D**) and propidium iodide (PI)-negative cells (**E**,**F**) were evaluated by flow cytometry 2 days after transduction. The gating strategy is available in Supplemental Figure S7. Data are expressed as the mean ± SD with technical replicates (n = 5) and ordinary one-way ANOVA with Tukey post-hoc test was conducted. **** *p* < 0.0001; ns, not significant.

After confirming the broad dual-tropic capacity of V/HN-LV particles and the possible synergistic mechanism of the VSV-G and SeV-HN envelope proteins, we investigated the potential of these LVs for ex vivo gene therapy. Thus, we transduced human cord blood samples with concentrated LV particles (produced as shown in Table S5) at 50,000 PP/cell, the major sources of human hematopoietic stem and progenitor cells (HSPCs). The HSCs and multipotent progenitor (MPP) cells were identified by the markers CD34, CD38, CD45RA, and CD90 (Figure S7). Flow cytometry analysis revealed that the percentage of GFP-positive cells was significantly improved in V/HN-LV or V/F/HN-LV transduced HSPCs compared with V-LV and V/F-LV (Figure 6A–D).

Moreover, the viability of V/HN-LV- or V/F/HN-LV-transduced cells was similar to that of V-LV- or V/F-LV-transduced cells, indicating that the addition of SeV envelope proteins to the LVs does not affect its virulence (Figure 6E,F). The results suggest that SeV-HN and VSV-G dual-pseudotyped LVs are applicable in an ex vivo clinical approach targeting human HSPCs.

## 4. Discussion

In dual-pseudotyped LVs, the viral envelope proteins are composed of two different heterologous envelope proteins that are phenotypically unmixed or mixed. Because each type of envelope protein has its own unique features, including receptor-binding specificity and intracellular trafficking, compatibility between envelope proteins must be considered to ensure proper LV particle assembly, functionality, and stability [53]. However, dual-pseudotyped LV particles have been more commonly developed using an unmixed phenotype, and studies characterizing direct interactions of envelope proteins with a mixed phenotype are scarce. In this study, we attempted to characterize VSV-G and wild-type SeV-HN phenotypically mixed heterologous dual-pseudotyped LV particles and optimize their production. We showed that these envelope proteins act synergistically, resulting in the broad dual-tropic ability of V/HN-LV particles with enhanced transduction efficiency.

We report that, in dual-pseudotyping, although the physical titers are similar, V/HN-LV particles showed improved biological titers compared with V-LV particles, whereas the V/F combination did not affect the biological titer. The consistent lack of functionality of wild-type SeV-F and/or SeV-HN pseudotyping for LVs confirmed the results of a previous report [28]. The amount of incorporated SeV-HN envelope protein in dual-pseudotyped V/HN-LV particles was increased while maintaining its functionality. This incorporation pattern was also observed for SeV-F, although infectivity was not affected, suggesting that VSV-G has some effects on the SeV envelope proteins during LV particle assembly. In addition, the combination of parainfluenza virus 5 (PIV5-F) protein and murine leukemia virus envelope proteins inhibited the infectivity of the dual-pseudotyped retrovirus (RV), confirming that the choice of envelope proteins and their compatibility are crucial for dual-pseudotyping [53]. By contrast, V/F-LV particles efficiently transduce mouse germline stem cells [38]. This is related to the high affinity between the asialoglycoprotein receptor (ASGP-R) on germline stem cells and the SeV-F protein [54,55]. These reports support our finding that the incorporation efficiency of the SeV wild-type envelope proteins is highly dependent on the VSV-G in the dual-pseudotyped LVs, and, most importantly, the wild-type SeV-HN envelope protein was correctly incorporated into the viral particles and also maintained its functionality. However, the interactions and intracellular trafficking of the envelope proteins that resulted in these positive outcomes are still unclear at the molecular level in the producer cells.

Using mouse, monkey, and human cell lines, and primary cultured cells, we demonstrated that the transduction efficiency of V/HN-LV was significantly higher than that of V-LV. In addition, it is noteworthy that the dual-pseudotyped LVs are also effective for use in HSPCs. Our viral tropism results indicated the potential gene delivery applicability of the V/HN-LV not only to basic science but also to ex vivo gene therapy targeting HSCs. Although our culture systems were limited to HSPCs, further investigation of the V/HN-LV infectivity of different cell types, including T cells and B cells that are relevant to gene therapy, may amplify its potential.

The careful balance of the two envelope proteins in viral particles to provide maximum effectiveness is crucial for dual-pseudotyped viral vectors. Moreover, despite the codon-optimization approach allowing for the synthesis of a large amount of protein using only a small amount of plasmid during the transient transfection, it has been less frequently used for viral envelope proteins [44]. We achieved technical optimization and showed that 0.1 unit of pCAG-khcHN (human codon-optimized SeV-HN with the Kozak sequence) plasmid vector per 1.0 unit of pCMV-VSV-G plasmid vector is adequate to maintain the balance of VSV-G and SeV-HN in the viral particles for V/HN-LV production. This suggests that codon optimization of the viral envelope protein is highly productive and facilitates the production of dual-pseudotyped LVs.

The negative charge on the surface of cells and viruses is due to terminal sialic acid residues. Cationic polymers, such as polybrene, which have been commonly utilized for LV experiments, increase viral transduction efficiency by counteracting repulsive electrostatic effects [56]. The VSV-G pseudotyped LV is covered with an N-glycosylation “shield” and its terminal sialic acids tightly adhere to each other promoting viral self-aggregation in the presence of Ca^2+^ ions [57,58]. The VSV-G pseudotyped LV treated with neuraminidase, which cleaves terminal sialic acid, showed increased infectivity compared with normal VSV-G [59]. Moreover, the HN envelope proteins of *Paramyxoviridae* family viruses remove sialic from progeny viruses to prevent self-aggregation [46,47]. We also confirmed that desialylation of VSV-G by SeV-HN glycoproteins in the V/HN-LVs is responsible for the improvement in infection efficiency. In this context, it is suggested that the desialylation of VSV-G improves viral transmission by preventing viral self-aggregation, reducing repulsive electrostatic effects, and promoting viral spread. In mammalian cells, the majority of the sialic acids are Neu5Ac and Neu5Gc, and the Neu5Gc is synthesized from Neu5Ac by the CMAH enzyme. However, humans cannot synthesize Neu5Gc because the human genome contains a deletion in the CMAH coding region [60]. Sialic acid type and linkage have an important effect on the infectivity of sialic acid-dependent viruses [45]. Our experiments, showing the effect of V/HN-LV infection on both desialylated HEK293FT cells and monkey CMAH-overexpressing HEK293FT cells, revealed that SeV-HN proteins preferentially utilize Neu5Ac to support viral entry into host cells. For influenza viruses, a similar pattern was reported, which was that Neu5Gc terminated glycoconjugates act as a decoy or low-binding receptor for the hemagglutinin (HA) domain [61,62]. We also observed that V/HN-LV transduction efficiency was decreased by neuraminidase inhibitor 2,3-dehydro-2-deoxy-N-acetylneuraminic acid (DANA) treatment, which suggests the relative contribution of the neuraminidase of SeV-HN for the viral entry. Hence, in addition to recognition of the LDL-R family by VSV-G [17], the V/HN-LV also acquired the capacity to enter cells by recognizing sialic acid, particularly Neu5Ac, on the host cell.

In summary, we performed a characterization and optimization study of phenotypically mixed heterologous dual-pseudotyped LVs and reported the possible synergistic mechanism of the VSV-G and wild-type SeV-HN envelope proteins that contribute to the improved infection efficiency of V/HN-LV. These envelope proteins act synergistically without interfering with their receptor-binding activity and confer on V/HN-LV a broad dual-tropic functional ability. Our results suggest the potential use of dual-pseudotyped V/HN-LV in various applications ranging from basic science to ex vivo human gene therapy. Taken together, although the dual-pseudotyping strategy is already well established, the current investigation implies that a compatible combination of VSV-G with another envelope protein can maintain the basic transduction efficiency of VSV-G while achieving specific cell targeting via an additional envelope protein.

## Data Availability

All data supporting this article are available in the Supplementary Files linked above.

## Supplementary Materials

The following supporting information can be downloaded at: https://www.mdpi.com/article/10.3390/v16060827/s1, Figure S1: Schematic illustration of the envelope proteins and gating strategy of flow cytometry; Figure S2: Post-infection images of the VSV-G and SeV envelope protein pseudotyped LV particles in a viral tropism assay; Figure S3: Images of LV transduced cells, and the titer of V/HN-LVs using the Kozak human codon-optimized SeV-HN plasmid; Figure S4: Mechanism of sialyltransferase inhibitor and lectin staining of desialylated cells; Figure S5: Functional assay of the desialylated LV particles; Figure S6: Effect of Neu5Ac and Neu5Gc treatment on LV transduction in HEK293FT cells; Figure S7: Gating strategy of hematopoietic stem and progenitor cells (HSPCs); Figure S8: Uncropped western blot images; Table S1: Production of LVs for VSV-G and SeV envelope protein pseudotyping; Table S2: Production of LVs for the viral tropism assay; Table S3: Production of LVs for VSV-G and SeV-HN envelope protein optimization experiments; Table S4: Production of LVs for sialic acid-related assays; Table S5: Production of LVs for the infection assay of HSPCs.
