# Supplementary material for: The Dual-Pseudotyped Lentiviral Vector with VSV-G and Sendai Virus HN Enhances Infection Efficiency through the Synergistic Effect of the Envelope Proteins
Source: Viruses. 2024 May 23;16(6):827. doi: 10.3390/v16060827 (PMC11209056; doi:10.3390/v16060827)
Supplement: Supplementary file 1 [file viruses-16-00827-s001.zip › Supplemental Figures S1-S8.pdf]

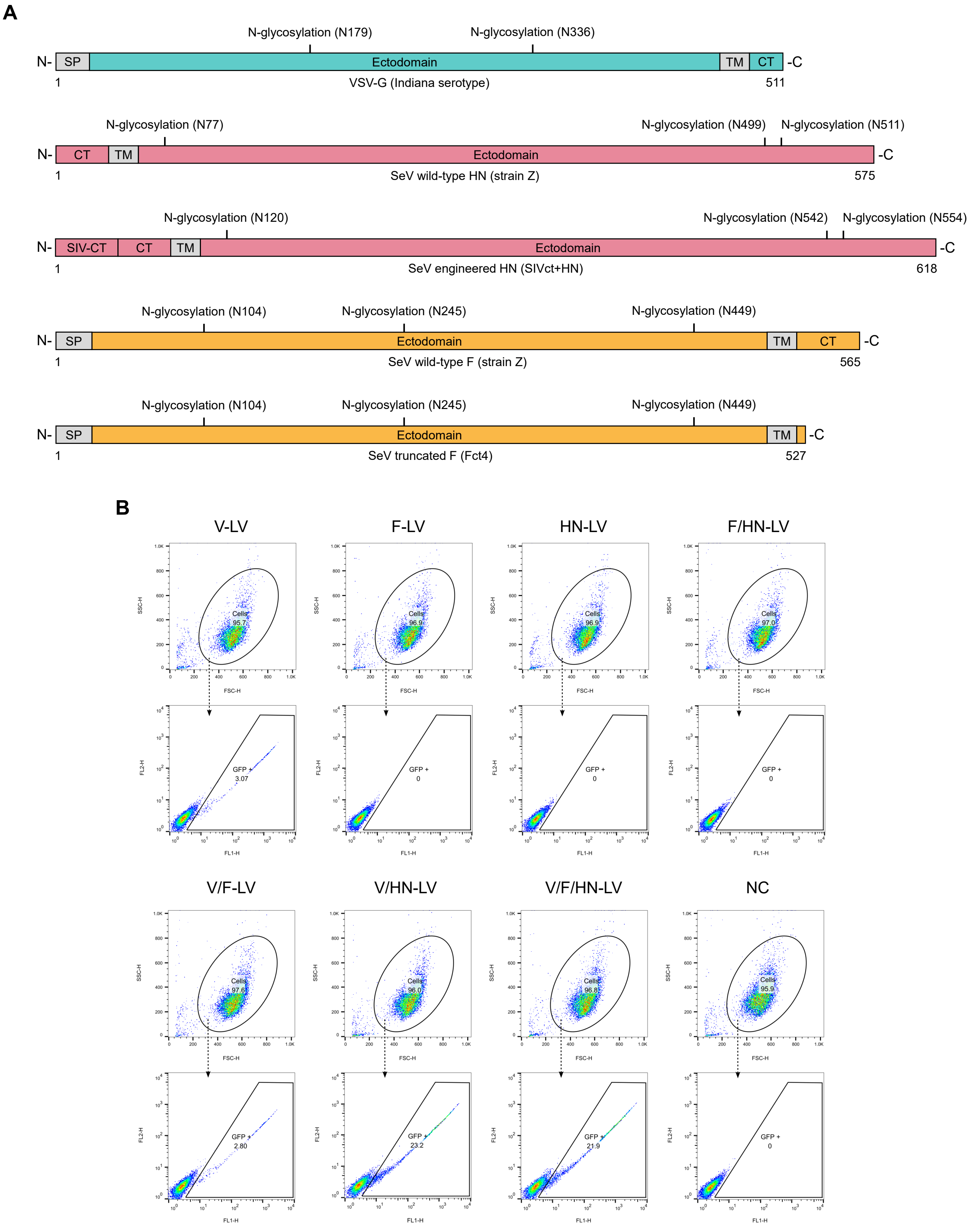

Figure S1. (A) Schematic illustration with turquoise represents VSV-G envelope glycoprotein, and the wild-type and engineered SeV-HN are shown in pale violet, as well as wild-type and truncated SeV-F are in yellow. The length of the envelopes, N-linked glycosylation sites, and positions of some specific regions are indicated from the N-terminus to the C-terminus. Signal peptide (SP), transmembrane domain (TM), cytoplasmic tail (CT), simian immunodeficiency viruses' cytoplasmic tail (SIV-CT). (B) Gating strategy of flow cytometry. Two days before analysis, HEK293FT cells were infected with LV particles at 200 PP/cell. The main cell population was gated from a forward scatter (FSC-H) versus side scatter (SSC-H) plot. Transduced GFP-positive cells can be detected on the right, outside of the negative cell population, in the FL1-H (530 nm) versus FL2-H (585 nm) plot.

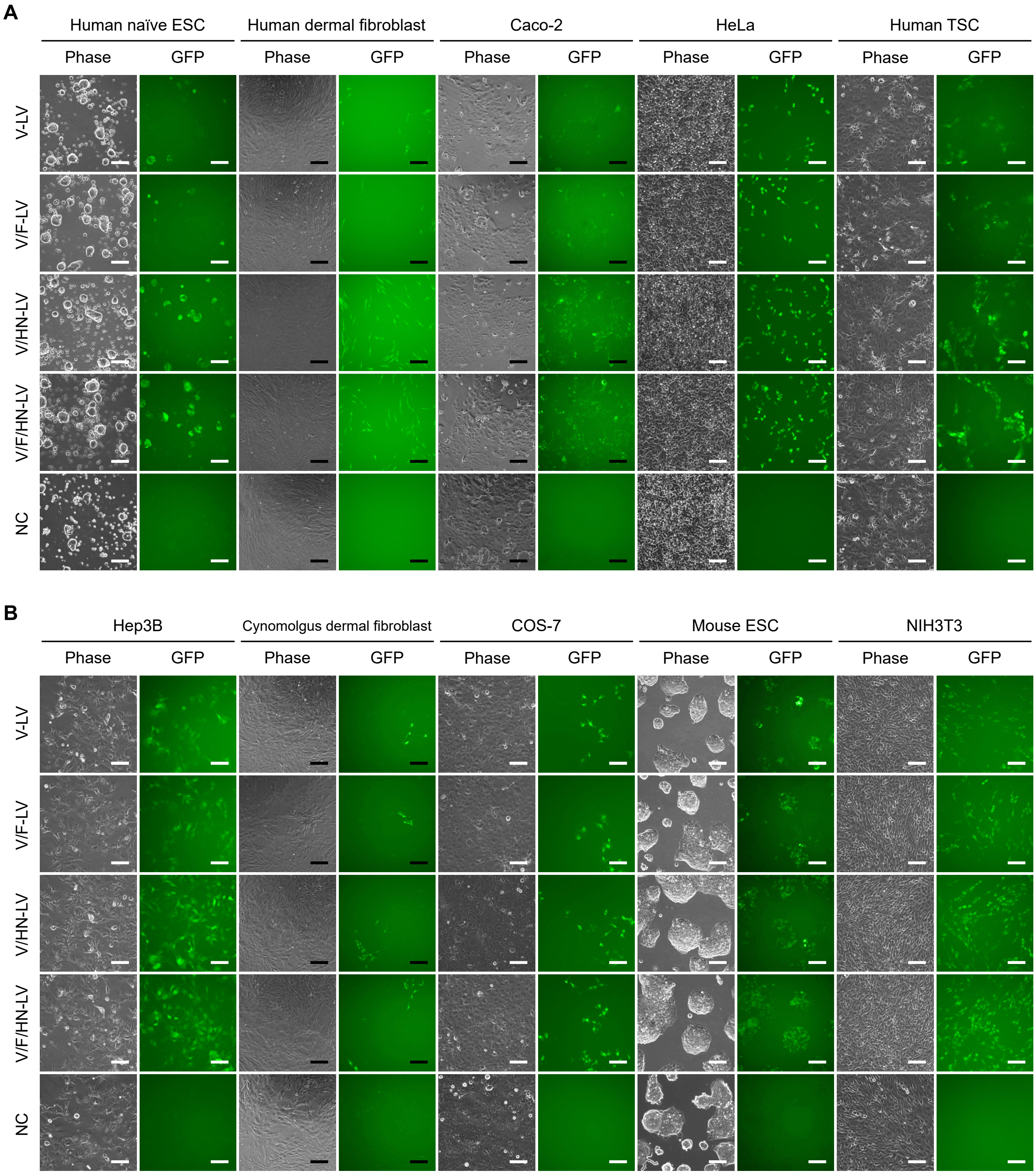

Figure S2. Post-infection images of the VSV-G and SeV envelope protein pseudotyped LV particles in a viral tropism assay. (A) Human naïve embryonic stem cells (ESCs) 4 days post-infection at 3200 PP/cell, human primary dermal fibroblast cells 4 days post-infection at 1600 PP/cell, colorectal adenocarcinoma cell line Caco-2 4 days post-infection at 3200 PP/cell, human cervical carcinoma derived cell line HeLa 3 days post-infection at 1600 PP/cell, and human trophoblast stem cells (TSCs) 3 days post-infection at 400 PP/cell. (B) Human hepatocellular carcinoma cell line Hep3B 4 days post-infection at 1600 PP/cell, cynomolgus monkey primary dermal fibroblast cells 4 days post-infection at 1600 PP/cell, African green monkey kidney fibroblast-like cell line COS-7 3 days post-infection at 1600 PP/cell, mouse embryonic stem cells (ESCs) 3 days post-infection at 3200 PP/cell, and mouse fibroblast cell line NIH3T3 3 days post-infection at 3200 PP/cell. Phase contrast and green fluorescent protein (GFP) images. Scale bar, 100 μm (white) and 200 μm (black).

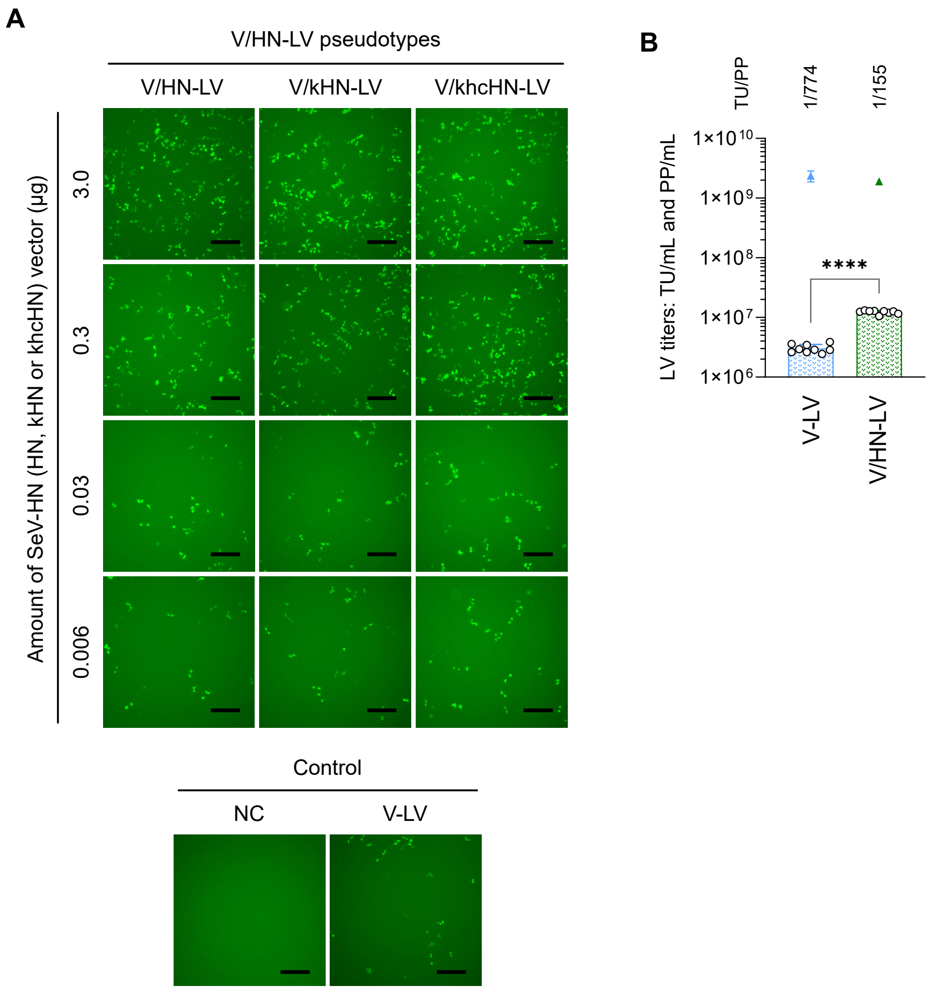

Figure S3. Images of LVs transduced cells, and the titer of V/HN-LVs using the Kozak human codon-optimized SeV-HN plasmid. (A) LV transduction of HEK293FT cells at 200 PP/cell, and GFP images 2 days post-infection. Scale bar, 200 µm. (B) The biological and physical titers of LV production that are detailed in Table S4, which were utilized in the experiments shown in Figure 4 and Figure 5. The bar graphs represent TU/ml, and triangles represent PP/ml. The ratio of TU to PP is shown above each set of LV titers. Data are expressed as the mean ± SD. Two-tailed T-test. ****$p<0.0001$.

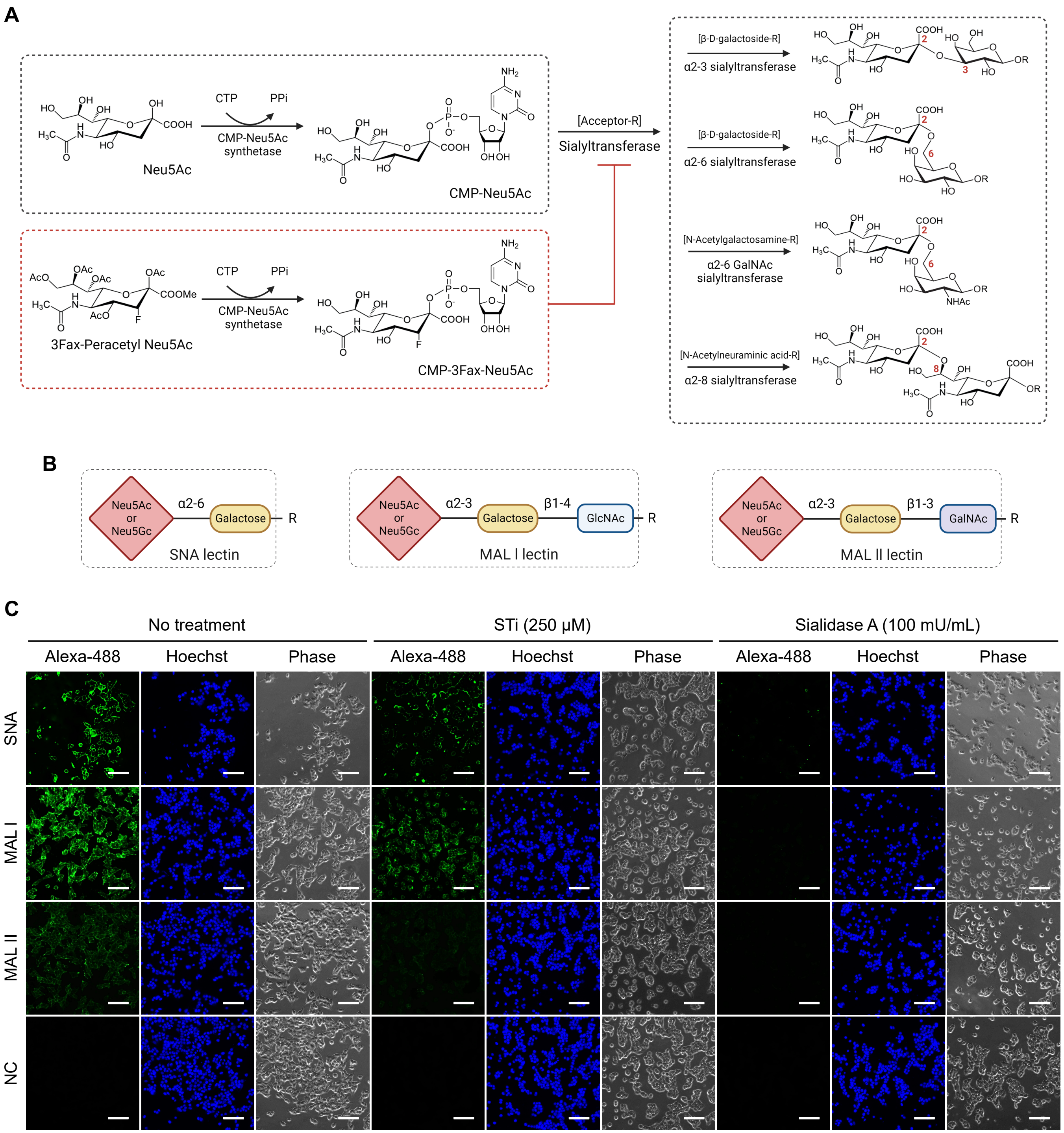

Figure S4. Mechanism of sialyltransferase inhibitor and lectin staining of desialylated cells. (A) The CMP-Neu5Ac synthetase (CMAS) enzyme can produce CMP-3Fax-Neu5Ac from the external 3Fax-Paracetyl Neu5Ac. Accumulation of CMP-3Fax-Neu5Ac in the cytoplasm inhibits endogenous sialyltransferase enzymes. (B) The Sambucus nigra agglutinin (SNA) lectin recognizes sialylated glycans with an α2-6 linkage, whereas Maackia amurensis (MAL I and MAL II) lectins identify an α2-3 sialylated glycosidic linkage. (C) Desialylation of the HEK293FT cells was confirmed by lectin staining. Biotinylated lectins were stained with Alexa fluor™ 488-conjugated streptavidin. Scale bar, 100 μm.

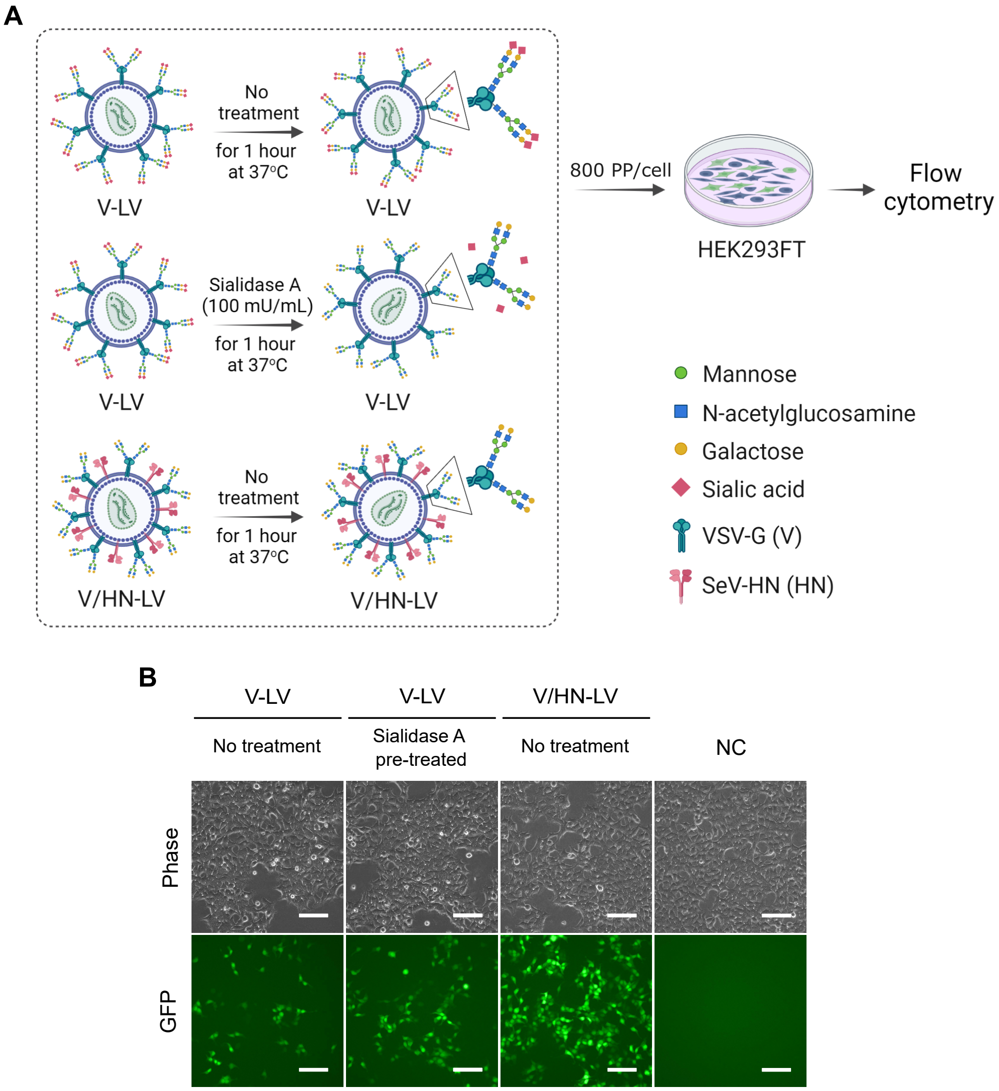

Figure S5. Functional assay of the desialylated LV particles. (A) Schematic diagram of the investigation of the effect of desialylation of VSV-G on LV transduction efficiency. V-LV was treated with sialidase A (100 mU/ml) for 1 h at 37°C. Untreated V-LV and V/HN-LV were included as negative and positive controls, respectively. Then, the LV particles were subjected to an infection assay on HEK293FT cells at 800 PP/cell. (B) Phase contrast and GFP images 2 days after infection in the experimental groups. Scale bar, 100 μm.

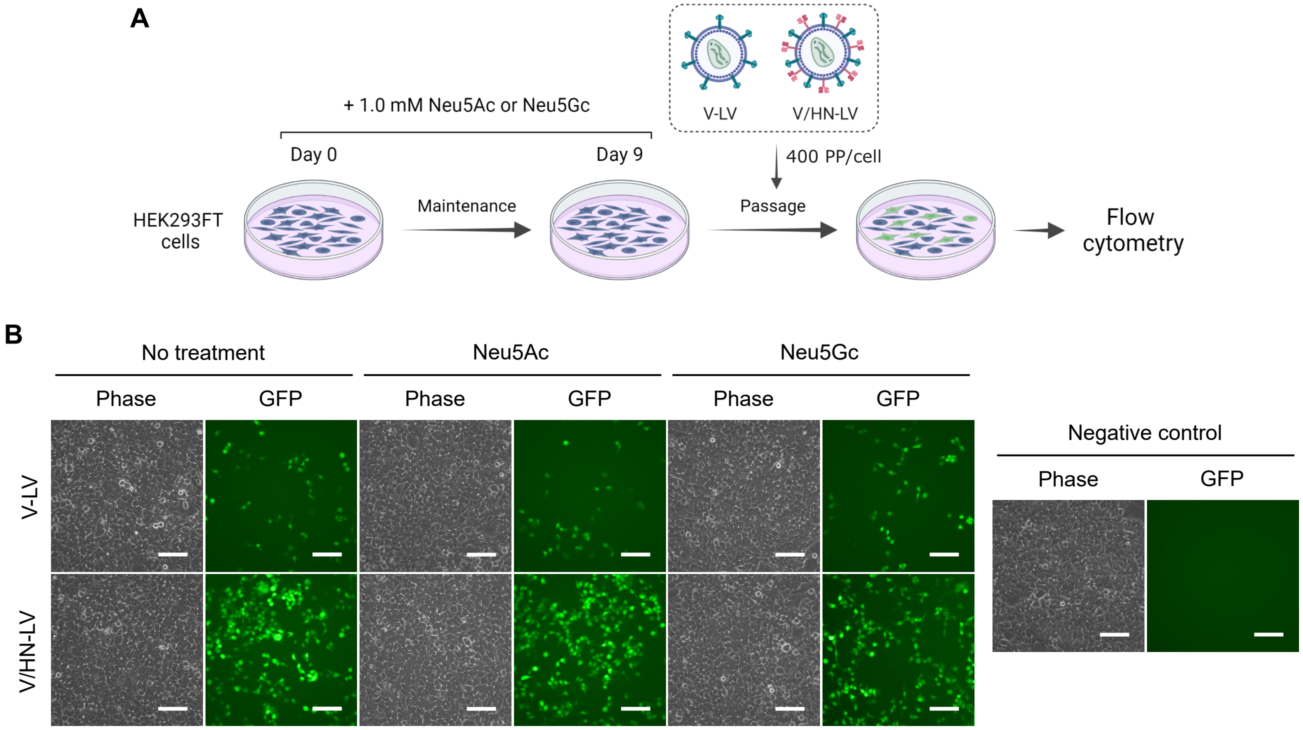

Figure S6. Effect of Neu5Ac and Neu5Gc treatment on LV transduction in HEK293FT cells. (A) Schematic diagram of the Neu5Ac and Neu5Gc feeding experiments in HEK293FT cells. The feeding of large amounts of Neu5Ac and Neu5Gc allow it to accumulate in the cell, thereby altering the cell surface sialic acid ratio. The cells fed with sialic acid were infected with LV particles at 400 PP/cell. (B) Phase contrast and GFP images 2 days after infection in the experimental groups. Scale bar, 100 μm.

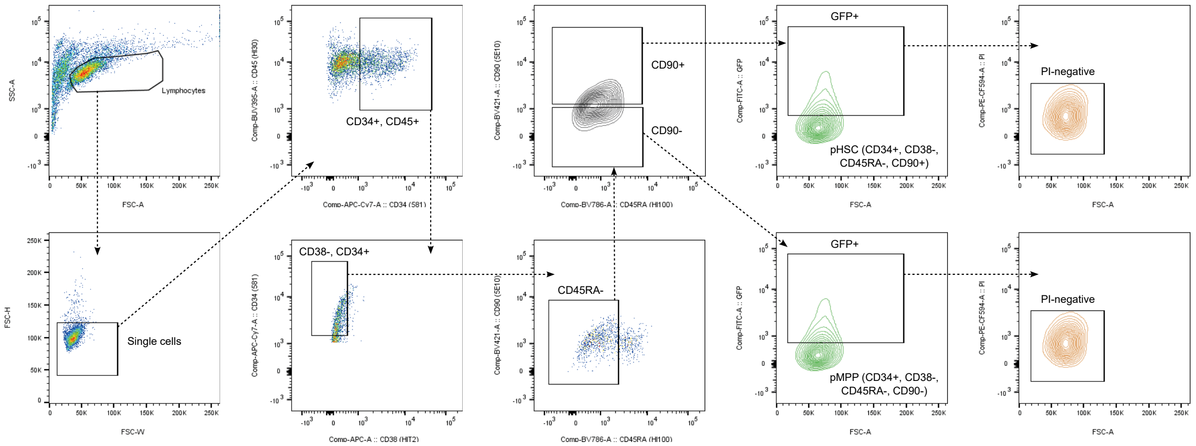

Figure S7. Gating strategy of hematopoietic stem and progenitor cells (HSPCs). The main cell population and debris were segregated in the FSC-A versus SSC-A plot, and the doublet cells were removed and the single cell population was gated in FSC-W versus FSC-H. CD34+, CD45+, CD38-, and CD45RA- cell populations were gated. Then, CD90+ cells (pHSC) and CD90- (pMPP) cells were gated and transduced GFP-positive cells can be detected at the top, outside of the negative cell population, in the FSC-A versus GFP plot. PI-negative cells were then detected in the GFP-positive population.

Figure 1E

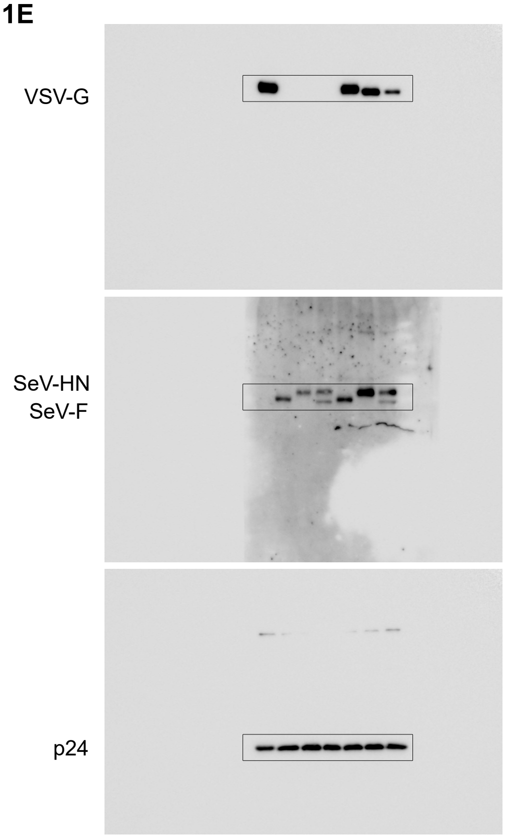

Figure 3D

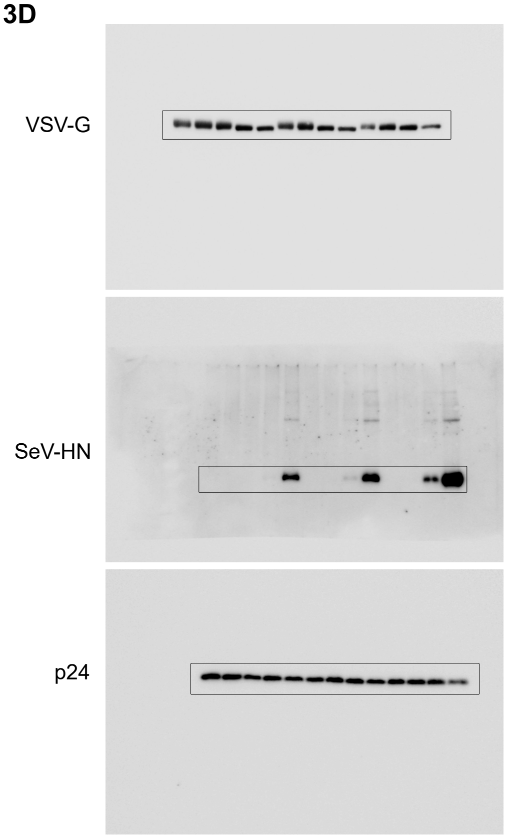

Figure 4F

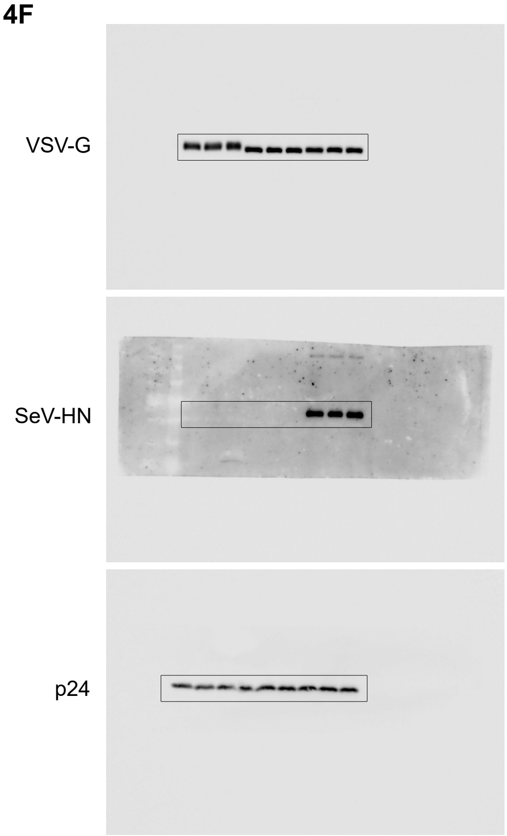

Figure S8. Uncropped western blot images. The uncropped western blot images used in Figure 1E, Figure 3D, and Figure 4F. The rectangle indicates the area shown in each figure panel.
