# Supplementary material for: The Dual-Pseudotyped Lentiviral Vector with VSV-G and Sendai Virus HN Enhances Infection Efficiency through the Synergistic Effect of the Envelope Proteins
Source: Viruses. 2024 May 23;16(6):827. doi: 10.3390/v16060827 (PMC11209056; doi:10.3390/v16060827)
Supplement: Supplementary file 1 [file viruses-16-00827-s001.zip › Supplemental Tables S1-S5.pdf]

Table S1. Production of LVs for VSV-G and SeV envelope protein pseudotyping

Plasmids and transfection reagents		LV pseudotypes						
		1	2	3	4	5	6	7
		VSV-G	SeV-F	SeV-HN	SeV-F/ SeV-HN	VSVG/ SeV-F	VSV-G/ SeV-HN	VSVG/ SeV-F/ SeV-HN
Plasmids	CS-CA-NLS-GFP (1.0 μg/μl)	6.0 μg	6.0 μg	6.0 μg	6.0 μg	6.0 μg	6.0 μg	6.0 μg
	pCAG-HIVgp (1.0 μg/μl)	3.0 μg	3.0 μg	3.0 μg	3.0 μg	3.0 μg	3.0 μg	3.0 μg
	pRSV-Rev (1.0 μg/μl)	1.5 μg	1.5 μg	1.5 μg	1.5 μg	1.5 μg	1.5 μg	1.5 μg
	pCMV-VSV-G (1.0 μg/μl)	1.5 μg	-	-	-	1.5 μg	1.5 μg	1.5 μg
	pCAG-F (1.0 μg/μl)	-	1.5 μg	-	1.5 μg	1.5 μg	-	1.5 μg
	pCAG-HN (1.0 μg/μl)	-	-	1.5 μg	1.5 μg	-	1.5 μg	1.5 μg
	pCAGGS (1.0 μg/μl) empty	3.0 μg	3.0 μg	3.0 μg	1.5 μg	1.5 μg	1.5 μg	-
	Total plasmids	15.0 μg	15.0 μg	15.0 μg	15.0 μg	15.0 μg	15.0 μg	15.0 μg
Buffer	HBSS (+)	900 μl	900 μl	900 μl	900 μl	900 μl	900 μl	900 μl
PEI	L-PEI 25K (1.0 μg/μl)	75.0 μg	75.0 μg	75.0 μg	75.0 μg	75.0 μg	75.0 μg	75.0 μg
Total transfection complex		990 μl	990 μl	990 μl	990 μl	990 μl	990 μl	990 μl

Note: Amount of the plasmids [4 plasmids system with 4:2:1 units ratio and 1 unit of VSV-G, SeV-F, and/or SeV-HN addition. The total amount is adjusted with empty vector] and PEI used for the LVs production in a 10 cm dish (V-LV, F-LV, HN-LV, F/HN-LV, V/F-LV, V/HN-LV, and V/F/HN-LV). 3 – 5 hours before transfection, when the cell confluence reached approximately 70%, the medium was replaced with exactly 15 ml of fresh complete medium. Transfection complexes were dropped wisely onto the cells. Then, 18 hours after transfection medium was replaced with 10 ml of fresh complete medium. Viral medium was harvested 2 days after the transfection.

Table S2. Production of the LVs for the viral tropism assay

Plasmids and transfection reagents		LV pseudotypes			
		1	2	3	4
		VSV-G	VSV-G/SeV-F	VSV-G/SeV-HN	VSV-G/ SeV-F/ SeV-HN
Plasmids	CS-CA-NLS-GFP (1.0 µg/µl)	5.0 µg	5.0 µg	5.0 µg	5.0 µg
	pCAG-HIVgp (1.0 µg/µl)	2.5 µg	2.5 µg	2.5 µg	2.5 µg
	pCMV-VSV-G-RSV-Rev (1.0 µg/µl)	2.5 µg	2.5 µg	2.5 µg	2.5 µg
	pCAG-F (1.0 µg/µl)	-	2.5 µg	-	2.5 µg
	pCAG-HN (1.0 µg/µl)	-	-	2.5 µg	2.5 µg
	pCAGGS (1.0 µg/µl) empty	5.0 µg	2.5 µg	2.5 µg	-
	Total plasmids	15.0 µg	15.0 µg	15.0 µg	15.0 µg
Buffer	HBSS (+)	900 µl	900 µl	900 µl	900 µl
PEI	L-PEI 25K (1.0 µg/µl)	75.0 µg	75.0 µg	75.0 µg	75.0 µg
Total transfection complex		990 µl	990 µl	990 µl	990 µl

Amount of the plasmids [3 plasmids system with 2:1:1 units ratio and 1 unit of SeV-F and/or SeV-HN addition. The total amount is adjusted with empty vector] and PEI used for the LVs production in a 10 cm dish (V-LV, V/F-LV, V/HN-LV, and V/F/HN-LV). 3 – 5 hours before transfection, when the cell confluence reached approximately 70%, the medium was replaced with 15 ml of fresh complete medium. Transfection complexes were dropped wisely onto the cells. Then, 18 hours after transfection medium was replaced with 10 ml of fresh complete medium. Viral medium was harvested 2 days after the transfection.

Table S3. Production of LVs for VSV-G and SeV-HN envelope optimization experiments

Plasmids and transfection reagents		LV pseudotypes												
		1	2	3	4	5	6	7	8	9	10	11	12	13
		VSV-G	VSV-G/SeV-HN				VSV-G/SeV-HN				VSV-G/SeV-HN			
Plasmids	CS-CA-NLS-GFP (1.0 μg/μl)	6.0 μg	6.0 μg	6.0 μg	6.0 μg	6.0 μg	6.0 μg	6.0 μg	6.0 μg	6.0 μg	6.0 μg	6.0 μg	6.0 μg	6.0 μg
	pCAG-HIVgp (1.0 μg/μl)	3.0 μg	3.0 μg	3.0 μg	3.0 μg	3.0 μg	3.0 μg	3.0 μg	3.0 μg	3.0 μg	3.0 μg	3.0 μg	3.0 μg	3.0 μg
	pCMV-VSV-G-RSV-Rev (1.0 μg/μl)	3.0 μg	3.0 μg	3.0 μg	3.0 μg	3.0 μg	3.0 μg	3.0 μg	3.0 μg	3.0 μg	3.0 μg	3.0 μg	3.0 μg	3.0 μg
	**pCAG-HN (1.0 μg/μl)**	-	**0.006 μg**	**0.03 μg**	**0.3 μg**	**3.0 μg**	-	-	-	-	-	-	-	-
	**pCAG-kHN (1.0 μg/μl)**	-	-	-	-	-	**0.006 μg**	**0.03 μg**	**0.3 μg**	**3.0 μg**	-	-	-	-
	**pCAG-khcHN (1.0 μg/μl)**	-	-	-	-	-	-	-	-	-	**0.006 μg**	**0.03 μg**	**0.3 μg**	**3.0 μg**
	pCAGGS (1.0 μg/μl) empty	3.0 μg	2.994 μg	2.97 μg	2.7 μg	-	2.994 μg	2.97 μg	2.7 μg	-	2.994 μg	2.97 μg	2.7 μg	-
	Total plasmids	15.0 μg	15.0 μg	15.0 μg	15.0 μg	15.0 μg	15.0 μg	15.0 μg	15.0 μg	15.0 μg	15.0 μg	15.0 μg	15.0 μg	15.0 μg
**Buffer**	HBSS (+)	900 μl	900 μl	900 μl	900 μl	900 μl	900 μl	900 μl	900 μl	900 μl	900 μl	900 μl	900 μl	900 μl
**PEI**	L-PEI 25K (1.0 μg/μl)	75.0 μg	75.0 μg	75.0 μg	75.0 μg	75.0 μg	75.0 μg	75.0 μg	75.0 μg	75.0 μg	75.0 μg	75.0 μg	75.0 μg	75.0 μg
**Total transfection complex**		990 μl	990 μl	990 μl	990 μl	990 μl	990 μl	990 μl	990 μl	990 μl	990 μl	990 μl	990 μl	990 μl

Amount of the plasmids [3 plasmids system with 2:1:1 ratio and variable amount of SeV-HN plasmid addition. The total amount is adjusted with empty vector] and PEI used for the LVs production in a 10 cm dish (V-LV, V/HN-LV, V/kHN-LV, and V/khcHN-LV). 3 – 5 hours before transfection, when the cell confluence reached approximately 70%, the medium was replaced with 15 ml of fresh complete medium. Transfection complexes were dropped wisely onto the cells. Then, 18 hours after transfection medium was replaced with 10 ml of fresh complete medium. Viral medium was harvested 2 days after the transfection.

Table S4. Production of LVs for sialic acid-related assay

Plasmids and transfection reagents		LV pseudotypes					
		VSV-G			VSV-G/SeV-HN		
		1	2	3	1	2	3
Plasmids	CS-CA-NLS-GFP (1.0 µg/µl)	8.0 µg	8.0 µg	8.0 µg	8.0 µg	8.0 µg	8.0 µg
	pCAG-HIVgp (1.0 µg/µl)	4.0 µg	4.0 µg	4.0 µg	4.0 µg	4.0 µg	4.0 µg
	pRSV-Rev (1.0 µg/µl)	2.0 µg	2.0 µg	2.0 µg	2.0 µg	2.0 µg	2.0 µg
	pCMV-VSV-G (1.0 µg/µl)	2.0 µg	2.0 µg	2.0 µg	2.0 µg	2.0 µg	2.0 µg
	pCAG-khcHN (1.0 µg/µl)	-	-	-	0.2 µg	0.2 µg	0.2 µg
	pCAGGS (1.0 µg/µl) empty	0.2 µg	0.2 µg	0.2 µg	-	-	-
	Total plasmids	16.2 µg	16.2 µg	16.2 µg	16.2 µg	16.2 µg	16.2 µg
Buffer	HBSS (+)	900 µl	900 µl	900 µl	900 µl	900 µl	900 µl
PEI	L-PEI 25K (1.0 µg/µl)	81.0 µg	81.0 µg	81.0 µg	81.0 µg	81.0 µg	81.0 µg
Total transfection complex (µl)		997.2	997.2	997.2	997.2	997.2	997.2

Amount of the plasmids [4 plasmids system with 4:2:1:1 units ratio and 0.1 unit of SeV-HN addition, or empty vector] and PEI used for the LVs production in a 10 cm dish (Biological triplicates of V-LV and V/HN-LV). 3 – 5 hours before transfection, when the cell confluence reached approximately 70%, the medium was replaced with 15 ml of fresh complete medium. Transfection complexes were dropped wisely onto the cells. Then, 18 hours after transfection medium was replaced with 10 ml of fresh complete medium. Viral medium was harvested 2 days after the transfection.

Table S5. Production of LVs for the infection assay of HSPCs

Plasmids and transfection reagents		Pseudotypes			
		**1**	**2**	**3**	**4**
		**VSV-G**	**VSV-G/SeV-F**	**VSV-G/SeV-HN**	**VSV-G/ SeV-F/ SeV-HN**
**Plasmids**	CS-CA-NLS-GFP (1.0 µg/µl)	18.0 µg	18.0 µg	18.0 µg	18.0 µg
	pCAG-HIVgp (1.0 µg/µl)	9.0 µg	9.0 µg	9.0 µg	9.0 µg
	pRSV-Rev (1.0 µg/µl)	4.5 µg	4.5 µg	4.5 µg	4.5 µg
	pCMV-VSV-G (1.0 µg/µl)	4.5 µg	4.5 µg	4.5 µg	4.5 µg
	pCAG-F (1.0 µg/µl)	-	4.5 µg	-	4.5 µg
	pCAG-HN (1.0 µg/µl)	-	-	4.5 µg	4.5 µg
	pCAGGS (1.0 µg/µl) empty	9.0 µg	4.5 µg	4.5 µg	-
	Total plasmids	45.0 µg	45.0 µg	45.0 µg	45.0 µg
**Buffer**	HBSS (+)	2700 µl	2700 µl	2700 µl	2700 µl
**PEI**	L-PEI 25K (1.0 µg/µl)	225.0 µg	225.0 µg	225.0 µg	225.0 µg
**Total transfection complex**		2970 µl	2970 µl	2970 µl	2970 µl

Amount of the plasmids [4 plasmids system with 4:2:1:1 ratio and 1 unit of SeV-F and/or SeV-HN addition. The total amount is adjusted with empty vector] and PEI used for the LVs production in a 15 cm dish (V-LV, V/F-LV, V/HN-LV and V/F/HN-LV). 4 – 5 hours before transfection, when the cell confluence reached approximately 70%, the medium was replaced with exactly 45 ml of fresh complete medium. Transfection complexes were dropped wisely onto the cells. Then, 18 hours after transfection medium was replaced with 25 ml of fresh complete medium. Viral medium was harvested 2 days after the transfection.
